# Quantifying Nanoparticle Layer Topography: Theoretical Modeling and Atomic Force Microscopy Investigations

**DOI:** 10.1021/acs.langmuir.3c02024

**Published:** 2023-10-12

**Authors:** Zbigniew Adamczyk, Marta Sadowska, Małgorzata Nattich-Rak

**Affiliations:** Jerzy Haber Institute of Catalysis and Surface Chemistry, Polish Academy of Sciences, Niezapominajek 8, 30-239 Krakow, Poland

## Abstract

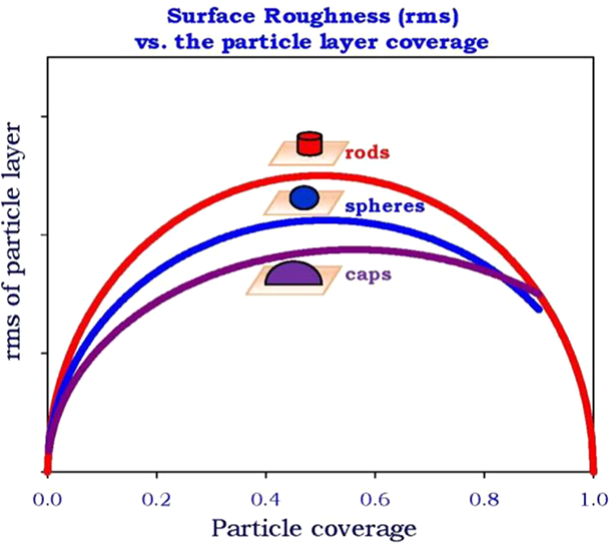

A comprehensive method consisting of theoretical modeling and experimental atomic force microscopy (AFM) measurements was developed for the quantitative analysis of nanoparticle layer topography. Analytical results were derived for particles of various shapes such as cylinders (rods), disks, ellipsoids, hemispheres (caps), etc. It was shown that for all particles, their root-mean-square (*rms*) parameter exhibited a maximum at the coverage about 0.5, whereas the skewness was a monotonically decreasing function of the coverage. This enabled a facile determination of the particle coverage in the layer, even if the shape and size were not known. The validity of the analytical results was confirmed by computer modeling and experimental data acquired by AFM measurements for polymer nanoparticle deposition on mica and silica. The topographical analysis developed in this work can be exploited for a quantitative characterization of self-assembled layers of nano- and bioparticles, e.g., carbon nanotubes, silica and noble metal particles, DNA fragments, proteins, vesicles, viruses, and bacteria at solid surfaces. The acquired results also enabled a proper calibration, in particular the determination of the measurement precision, of various electron and scanning probe microscopies, such as AFM.

## Introduction

Particle deposition at solid surfaces leading to the formation of self-assembled layers is important for many practical processes such as water filtration,^1,2^ flotation,^3,4^ coating formation,^5^ paper making,^6^ microelectronics, and colloid lithography.^7−10^ Silver particle layers find applications in chemical analysis,^11−13^ catalysis,^14−16^ and cosmetic pharmaceutical and textile industries.^17,18^ Similarly, gold nanoparticle assemblies are applied for the preparation of electrochemical,^19−23^ plasmonic,^24−26^ and piezoelectric sensors^27^ for catalytic purposes^28−31^ and in advanced physical processes.^32,33^

Analogously, a controlled deposition of bioparticles, such as protein molecules, viruses, bacteria, and cells, is necessary for their efficient separation by chromatography and filtration, for biosensing and immunological assays, etc. Determining the attachment of viruses to abiotic substrates (e.g., metals) is essential for devising strategies for their efficient deactivation and removal.

One should consider that the shape of noble metal particles,^34^ carbon nanotubes,^35−37^ silica particles,^38−41^ and synthetic polymer microparticles^42−44^ is often anisotropic, resembling prolate spheroids or cylinders (rods). The anisotropic molecule shape is common among biocolloids such as DNA fragments,^45−47^ proteins,^48,49^ viruses,^50,51^ and bacteria,^52,53^ for example the *E. coli* strain.

Because of its significance, particle and bioparticle deposition has been extensively studied using a variety of techniques, such as reflectometry and ellipsometry,^54,55^ optical waveguide lightmode spectroscopy (OWLS),^56−58^ surface plasmon resonance (SPR),^59^ quartz crystal microbalance (QCM),^60−64^ and electrokinetic methods such as the streaming potential.^65^ However, these are mostly indirect techniques requiring proper calibration in order to yield the absolute data. The calibration can be effectively carried out using optical microscopy for the microparticle size range^65^ or scanning electron microscopy (SEM) in the case of nanoparticles.^66,12^ Using the particle layer images derived from these techniques, their coverage can be determined by a direct counting procedure. However, this can become rather inaccurate in the case of SEM, where the sputtering of a subsidiary conductive layer is typically applied, which can modify the particle size and shape, eliminating the possibility of a proper topographical analysis. In this respect, the most versatile is atomic force microscopy (AFM)^63,67−69^ comprising its high-speed version,^70−72^ particularly suited for bioparticles, which directly furnishes the three-dimensional information about the particle layer topography. Such an analysis, comprising the calculation of the rms factor characterizing the magnitude of the surface roughness, was carried out for gold^69^ and polymer nanoparticle^73^ layers on silica. However, it should be considered that the AFM method itself has some limitations, mainly stemming from the finite size of the tip leading to the convolution effects^74^ and from the discretization of the scanning area.^75^ Although the significance of these effects can be decreased by selecting high-quality tips and properly adjusting the scan area to the particle size, the lack of reference theoretical data prohibits a quantitative estimation of the precision of the AFM measurements. Few exemptions represent the work of Batys and Weroński,^76^ who numerically calculated the average height and the rms of spherical particle multilayers on a solid substrate. In ref (73), the rms factor of the spheroidal particle layers was analytically calculated as a function of the coverage.

Given the deficit of topographical data, the main goal of this work was to develop a comprehensive description of the particle layer topography comprising such parameters as the average height, the rms, the skewness characterizing the roughness asymmetry, and the kurtosis. Analytical results are derived for particles of various shapes such as cylinders (rods), disks, parallelepipeds, ellipsoids, spheroids, spheres, caps of various shapes, etc. These results, valid for the entire range of particle coverage, are compared with computer modeling performed according to the Monte Carlo random sequential adsorption (RSA) approach. The role of the discretization pertinent to the AFM measurements is quantitatively evaluated. The applicability of the theoretical approach for the interpretation of the experimental data derived from AFM measurements for polymer particle layers on mica and silica is confirmed.

It is argued that our results can directly be used for a topographical characteristization of the surface assemblies of carbon nanotubes, silica and polymer particles, macroion microstructures, DNA chains, proteins, viruses, and bacteria. One can also expect that the results can serve for the prediction of the rms and other topographical parameters of protein and nanoparticle layers (coronas) on curved interfaces, widely studied in the literature but inadequately interpreted because of the lack of appropriate experimental techniques.

Additionally, the acquired results enable proper calibration, in particular the determination of the measurement precision, of various electron and scanning probe microscopies, such as AFM.

## Theoretical Section

### Quantifying the Topography of Particle-Covered Surfaces

The central moments of a rough surface μ_*q*_ are given by the general formula
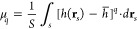
1where *q =* 2,3,4···, *S* is the projection area of the surface, *h*(**r**_*s*_) is the local height of the surface profile measured relatively to a reference planar surface located at *h*_0_, **r**_*s*_ is the surface position vector, and *h̅* is the average height of the surface calculated as
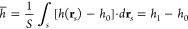
2where 

It should be noted that the average height is not unique by definition because it depends on the position of the reference plane. In contrast, the central moments are uniquely defined because μ_*q*_(*h* – *h*_0_) = μ_*q*_(*h*), therefore they are independent of the location of the reference plane.

The second, third, and fourth moments can be transformed to the useful forms
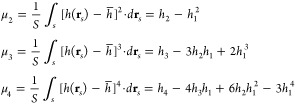
3where the surface integrals *h*_2_–*h*_4_, are given by
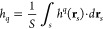
4where *q =* 2,3,4.

Consequently, the basic topographical parameters such as the root-mean-square (*rms*), the skewness (*sk*), and the kurtosis (*ku*) of a rough surface can be calculated as follows
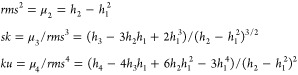
5

If the roughness stems from the presence of surface features (particles) attached to a planar surface located at *h* = 0, one can explicitly express these topographical parameters in the useful analytical form discussed below.

### Modeling of Particle Layer Formation

Computer experiments aimed at the determination of the above-defined topographical parameters were carried out according to the algorithm comprising the following steps:

(i) creation of the particle layer of a desired coverage applying a Monte-Carlo type, coarse grained approach,
(ii) quantitative characteristics of the layer in terms of the radial distribution function,
(iii) superimposition of a rectangular net over the particle layer characterized by the mesh size within the range pertinent to the AFM scans, and
(iv) calculation of the topographical parameters for the discrete set of points resulting from the mesh point distribution.

For the creation of the particle layer, the extended random sequential adsorption modeling (referred to as soft-RSA) was applied considering the electrostatic interactions of the incoming particle with all previously attached particles to the surface. The calculation algorithm was based on the following rules:^77−81^ a virtual molecule of a fixed size was generated, whose position within the adsorption domain was selected at random; if the particle fulfilled the defined adsorption criteria, it was treated as firmly attached to the surface, and thus its position was unchanged during the entire process; if the adsorption criteria were not fulfilled, a new adsorption attempt was made, uncorrelated with the previous ones. Two necessary deposition criteria were adopted: (i) no overlapping of the virtual particle with others attached to the surface and (ii) the availability of an uncovered surface area on the substrate surface large enough to accommodate the virtual particle.

Because of the simplicity of governing rules, this RSA algorithm enabled the generation of large populations of deposited particles. This enabled us to attain the precise theoretical calculations of the topographical parameter better than 0.1%.

The interaction energy of the virtual particle ϕ_*v*_ was calculated by summing up the pair potentials within the interaction zone expressed using the linear superposition approach.^81^

## Experimental Section

### Materials and Methods

All chemical reagents comprising sodium chloride, sodium hydroxide, and hydrochloric acid were commercial products of Sigma-Aldrich and were used without additional purification. Ultrapure water was obtained using the Milli-Q Elix & Simplicity 185 purification system from Millipore.

The stock suspensions of positively charged amidine and negatively charged sulfate polystyrene microparticles (latexes) were supplied by Invitrogen. These suspensions of a concentration determined by densitometry and the dry mass method were diluted to the desired concentration, typically 10–500 mg L^–1^, before each adsorption kinetic measurement. The ionic strength of the suspensions was adjusted by the addition of a NaCl solution, and the pH was regulated by the addition of hydrochloric acid solutions.

As a model substrate for performing the adsorption studies, muscovite mica and silica sensors were used. The mica sheets were freshly cleaved and used without further pretreatment in each set of experiments. Quartz/silicon dioxide (SiO_2_) sensors used in the experiments were supplied by Q-Sense, Gothenburg, Sweden. Before every measurement, the sensors were cleaned in a mixture of 96% sulfuric acid (H_2_SO_4_), hydrogen peroxide (30%), and ultrapure water in a volume ratio of 1:1:1 for 3 min. Afterward, the sensors were rinsed with deionized water at 80 °C for 30 min and dried out in a stream of nitrogen gas. The roughness of the sensors was examined by the semicontact mode of an atomic force microscopy (AFM) imaging carried out under ambient conditions.

The diffusion coefficient of particles was determined by dynamic light scattering (DLS) using the Zetasizer Nano ZS instrument from Malvern. The hydrodynamic diameter was calculated by using the Stokes–Einstein relationship. The particle size distribution was independently determined by laser diffractometry using the LS 13 320 Beckman Coulter device, which furnishes precise size distribution.

The electrophoretic mobility of particles was measured using the laser Doppler velocimetry (LDV) technique with the same apparatus. The zeta potential was calculated using the Ohshima equation, considering the ion polarization effect.^82^

The rms and other topographical parameters of the particle layers were determined by the ex situ AFM method. Accordingly, the adsorption kinetic runs were stopped after discrete time intervals and the mica sheets were removed from the suspension and imaged under ambient conditions using the NT-MDT Solver BIO device with the SMENA SFC050L scanning head. The number of particles per unit area (typically one square micrometer), denoted hereafter by *N*, was determined by a direct counting of over a few equal-sized areas randomly chosen over the sensor with the total number of particles of about 2,000. The rms, skewness, and kurtosis of the layers were calculated using Gwyddion software.

The zeta potential of mica was determined via streaming potential measurements performed according to the procedure described in ref (83) applying the Smoluchowski formula, where the correction for the surface conductivity was considered.

All experiments were performed at a temperature of 298 K.

## Results and Discussion

### Calculations of the Topographical Parameters

If the roughness originates from the assembly of *N*_p_ surface features (particles) of equal size and the same shape, the average height and the remaining topographical parameters can be calculated from eq 5, evaluating the surface integrals defined by eq 4. As a result, one obtains the following expressions (Supporting Information):
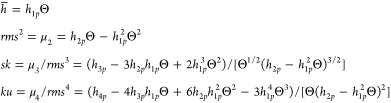
6where
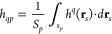
7*q =* 1, 2,3,4, *S_p_* is the characteristic cross-sectional area of the particle, and
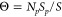
8is the absolute particle coverage.

It is worth mentioning that eq 6 is valid for arbitrary coverage and distribution of particles within the assembled layer. It is also applicable for any particle shape where the double integrals can be evaluated either analytically or by numerical methods. A simple analytical expression can be obtained for the category of particles characterized by the cross-sectional area independent of the coordinate perpendicular to the planar surface and the side walls perpendicular to the surface such as cylinders of arbitrary, e.g., of elliptic, cross-sectional area, disks, parallelepipeds (cubes), etc. For these particles, the double integrals defined by eq 7 can be immediately evaluated because *h*(**r**_*s*_) is independent of the position vector. In consequence, the and *h*_1*p*_ to *h*_4*p*_ coefficients become
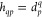
9where *d_p_* is the characteristic particle dimension perpendicular to the surface (see Table 1).

**Table 1:**
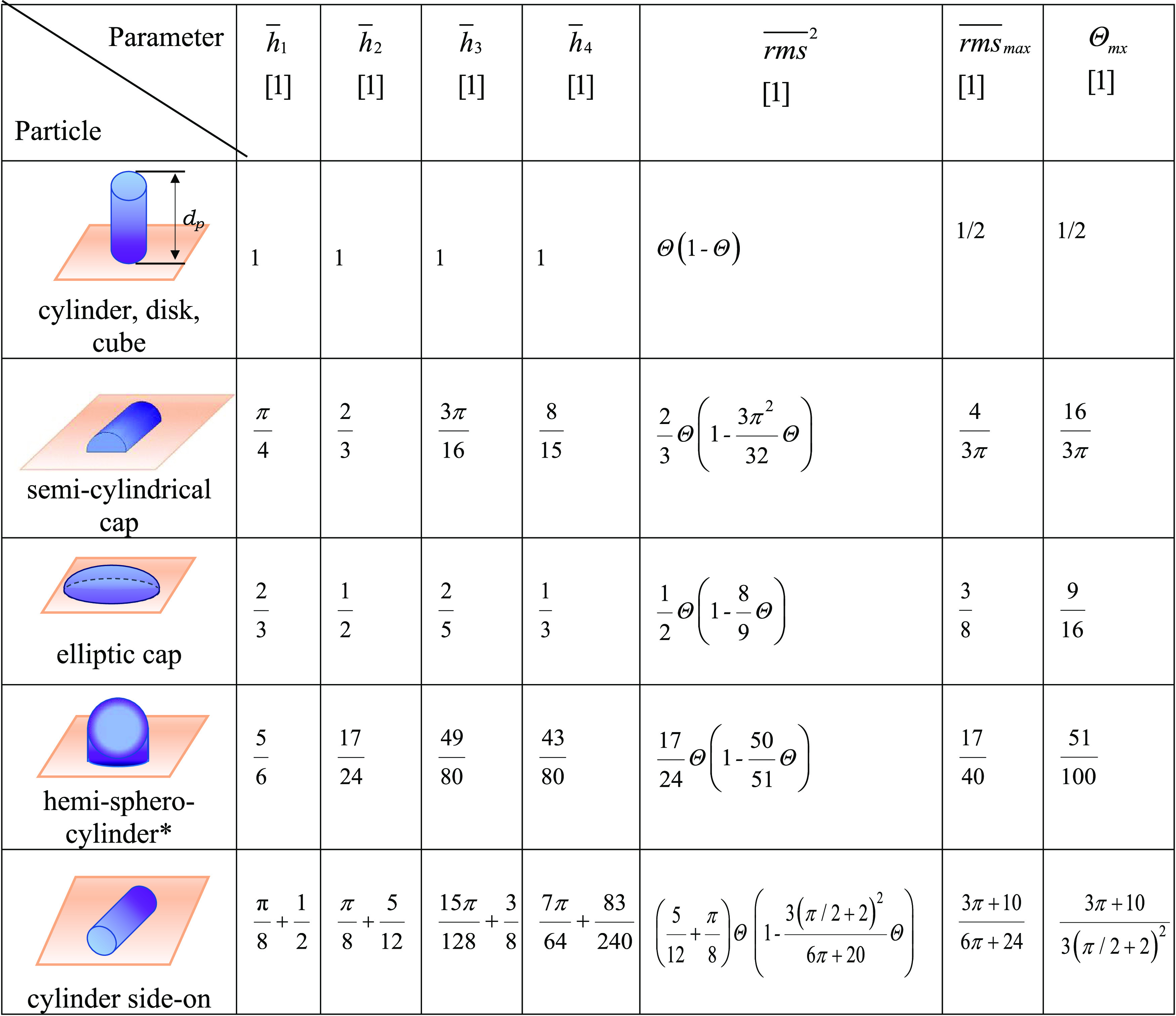
The Normalized Coefficients Characterizing the Topography of Surfaces Covered by Particles of Various Shape Analytically Derived from S7 (Supporting Information)^a^

a **Footnotes, definitions:**; ; *d_p_* - particle dimension perpendicular to the interface, Θ = *N*_*p*_*S*_*p*_/*S* - absolute particle coverage, *N*_*p*_ - number of particles in the layer, *S_p_* - particle cross-sectional area (at the interface);  (maximum  of the particle layer);  (coverage of the  maximum). *The case of the elliptic hemisphero-cylinder topographically corresponds to the ellipsoidal particles, comprising spheroids and spheres.

In this case, according to eq 6, the average height, the root-mean-square (*rms*), the skewness (*sk*), and the kurtosis (*ku*) of the particle layer are given by
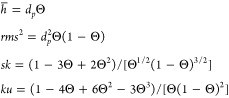
10

The *h*_1*p*_ to *h*_4*p*_ coefficients were also calculated for the particles in the form of elliptic caps, hemisphero-cylinders, and cylinders aligned parallel to the interface, evaluating the corresponding double integrals in the Cartesian coordinate system (Supporting Information). For the sake of convenience, these coefficients were normalized as follows
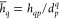
11

Using these coefficients, the topographical parameters can be expressed as follows
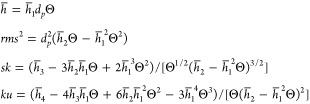
12

The *h̅*_1_–*h̅*_4_ coefficients calculated for various particle shapes (Supporting Information) are collected in Table 1, where the maximum values of the normalized *rms*/*d_p_* are also given as well as the coverage where the maximum occurs.

Analyzing these results, one can note that the normalized topographical coefficients *h̅*_1_ to *h̅*_4_ for all particle shapes are markedly lower than those for cylindrical particles, where they all attained the maximum value of unity. Thus, for elliptic caps and hemisphero-cylinders (spheres), one has *h̅*_1_ = 2/3 and 5/6, respectively, whereas *h̅*_4_ = 1/2 and 17/24, respectively. In consequence, the maximum normalized  values are equal to 1/2, 3/8, and 17/40 for cylinders, caps, and spheres, respectively.

It should be mentioned that for all feature shapes, the average layer height and the rms scale up linearly with the characteristic particle dimension (perpendicular to the surface), whereas the skewness and kurtosis only depend on the coverage (see eq 12). The latter property has interesting practical repercussions, enabling a facile determination of the particle layer coverage exploiting the skewness acquired from experiments.

Moreover, using eq 12, one can formulate the limiting expressions valid low coverage range where Θ ≪ 1, which assume the following form
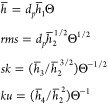
13

Thus, under this regime, the average layer height linearly increases with the particle coverage, the rms is proportional to the square root of the coverage, and the skewness is proportional to Θ^–1/2^. This indicates that the sensitivity of the latter parameter to particle coverage is exceptionally large, creating the possibility of precise measurements.

One can also expect that the topographical parameters collected in Table 1 can be related to the parameters used to express the roughness of mounded surfaces, e.g., of the QCM sensors.^84−86^ This comprises the correlation length denoted by ξ and the wavelength denoted by λ.^87^ Thus, for spherical particle layers, these parameters can be approximated by
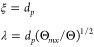
14where Θ_*mx*_ is the maximum coverage of the particle layer equal to 0.547 for the RSA regime and 0.785 and 0.907 for the regular and hexagonal closely packed particle layers, respectively.^81^

From eq 14, one can also predict that Θ = Θ_*mx*_(ξ/λ)^2^. In consequence, the limiting expressions given by eq 13 become
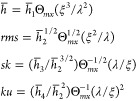
15

Although the above-discussed results are strictly valid for monodisperse particle layers, one can expect that they can also be used for the estimation of the topological parameters of clusters formed, for example, by the alternating deposition of oppositely charged particles.^88^ However, implementation of our formulae would require to know not only the coverage but also the structure of the formed clusters, or at least their shape and the maximum height.

**Figure 1:**
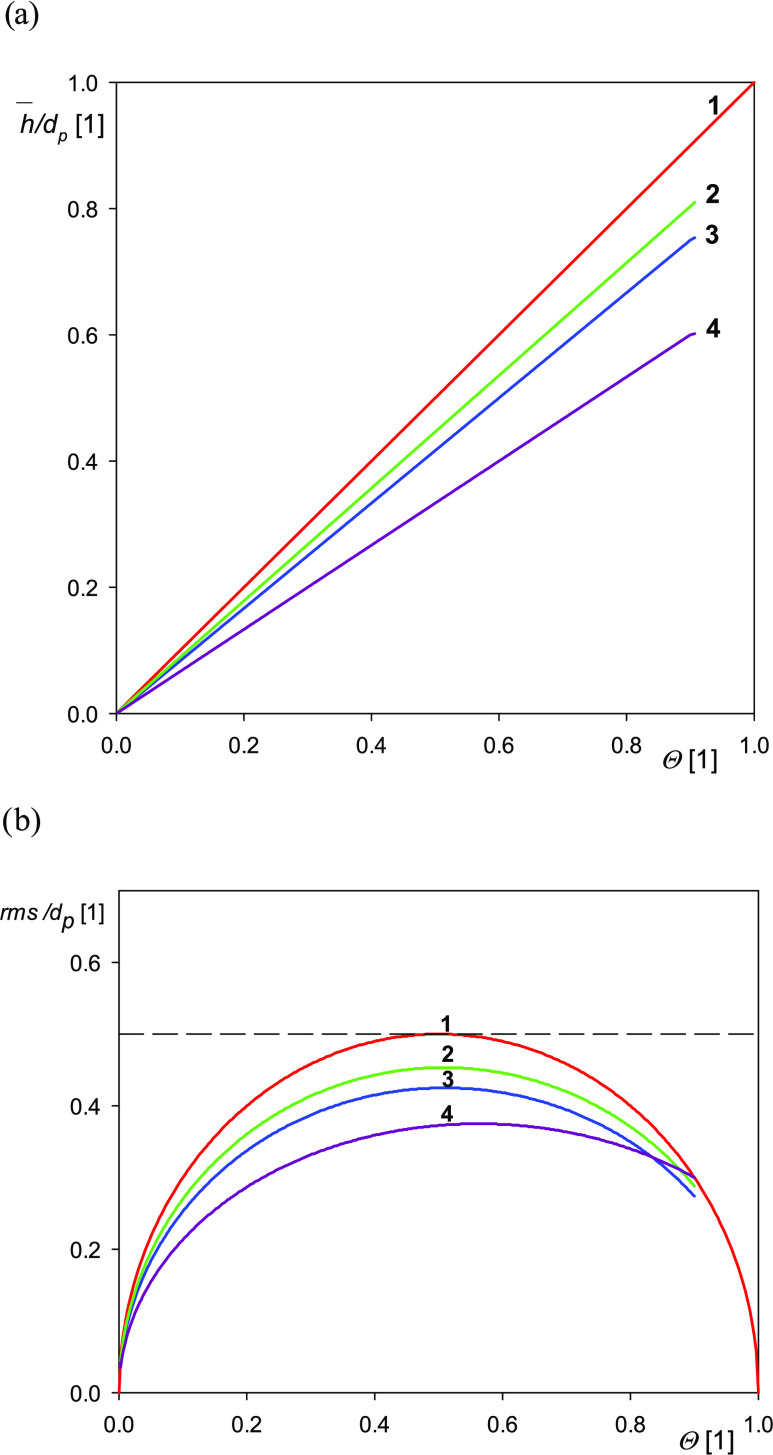
(a) Dependence of the normalized layer height on the coverage Θ calculated from eq 12. (b) Dependence of the normalized layer *rms*/*d_p_* on the coverage Θ calculated from eq 12. (1) Cylinders, end-on, disks, parallelepipeds, cubes. (2) Cylinders, side-on. (3) Elliptic sphero-cylinders (spheres), ellipsoids, spheroids. (4) Ellipsoidal caps, hemispherical caps.

**Figure 2:**
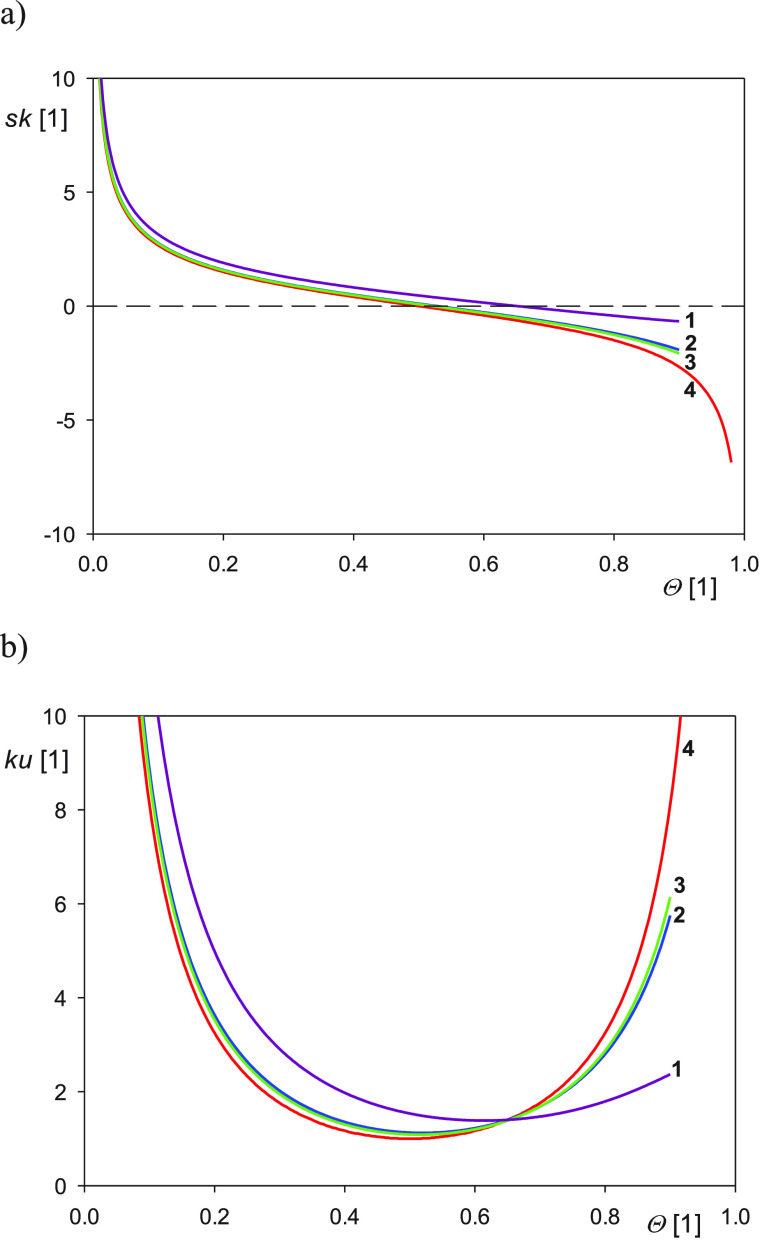
(a) Dependence of the particle layer skewness on the coverage Θ calculated from eq 12. (b) Dependence of the particle layer kurtosis on the coverage Θ calculated from eq 12. (1) Ellipsoidal caps, semispherical caps. (2) Elliptic sphero-cylinders, ellipsoids, spheroids, spheres. (3) Cylinders, side-on. (4) Cylinders, end-on, disks, parallelepipeds, cubes, etc.

Dependencies of the topographical parameters on the particle coverage calculated from eq 12 are graphically shown in Figures 1 and 2. It is worth mentioning that the relationships shown in these figures are independent of the structure of the particle layer.

In Figure 1a, the normalized particle layer height is plotted vs the coverage for cylinders of an arbitrary cross-sectional area under different orientations, for parallelepipeds, for elliptic hemisphero-cylinders, and for ellipsoidal caps. Interestingly, the two latter cases also comprise the spherical and hemispherical particles, respectively. It should be mentioned that in the case of particle layers, the calculation of the average height is unique because the reference height corresponds to the location of the planar surface, assumed to be at *z* = 0. As can be seen in Figure 1a in all cases, the normalized height linearly increases with the coverage and the maximum slope of unity is attained for cylinders, whereas it is equal to 5/6 for spheres and 2/3 for hemispherical caps, respectively.

In Figure 1b, the dependence of the normalized *rms*/*d_p_* on the coverage for various particle shapes is shown. As can be seen, in contrast to the average layer height, all of these dependencies are nonmonotonic and are characterized by a maximum occurring at the coverage about 0.5. Interestingly, for the cylinders (parallelepipeds), the dependence of the *rms* on the coverage is described by a hemicircle with its origin at Θ = 0.5 and the radius of 0.5. Slightly more asymmetric dependencies are also obtained for other particle shapes showing that a given *rms* value can be obtained for two different coverages of the particle layer; hence, the *rms* vs Θ relationships are not unique.

It is also worth mentioning that the normalized *rms*/*d*_*p*_ abruptly increases for low particle coverage but never exceeds the value of 0.5. This is described in the case of spheroids and spheres by the explicit expression^73^
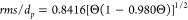
16

One can calculate from eq 16, which shows that for Θ = 0.1, the *rms*/*d*_*p*_ = 0.253, which is almost two times smaller than the maximum value 0.425 occurring at Θ = 0.51. Analogously, at the maximum coverage equal to 0.547 pertinent to the RSA regime, the *rms*/*d*_*p*_ = 0.424. For the regular and hexagonal layers of spheres, where Θ_*mx*_ = 0.785 and 0.907, respectively, the *rms*/*d*_*p*_ decreases to 0.358 and 0.267, respectively.

**Figure 3:**
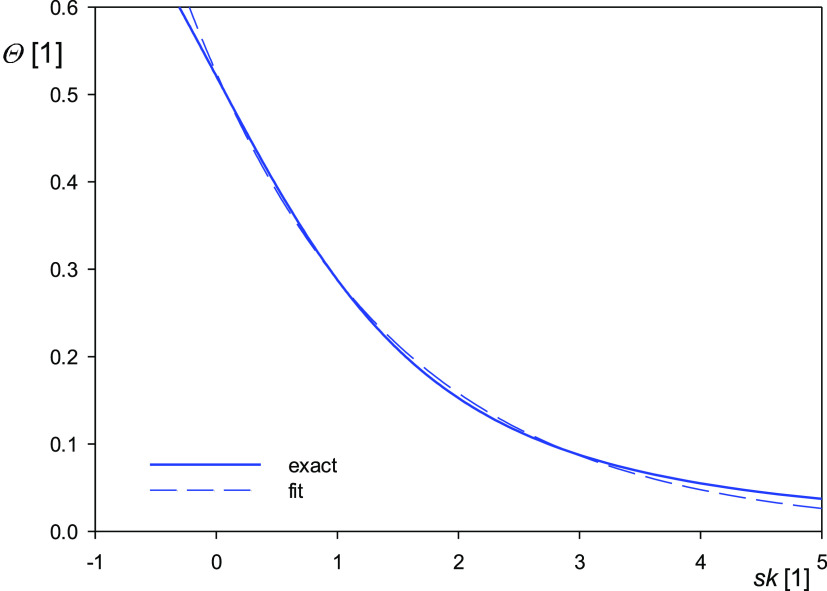
Dependence of the coverage of spherical particles Θ calculated by the inversion of eq 12 on the layer skewness. The dashed line shows the results calculated using the fitting function: 0.53 exp(−0.60*sk*).

In Figure 2a,b, the dependencies of the layer skewness (characterizing the height distribution asymmetry) and the kurtosis (characterizing the curvature of these distributions around the maximum) are shown. It can be seen that the skewness for all particle shapes remains a monotonic function of the coverage, whereas the kurtosis, analogously as the layer *rms*, is a nonmonotonic function exhibiting a minimum at Θ ca. 0.5–0.6. It is also interesting to observe that the differences in the skewness among various particle shapes are practically negligible for coverages below 0.5. Therefore, this property of the skewness parameter can be used for a facile determination of the particle coverage even if their shape and size are not known. In order to confirm this point more explicitly, the expression for the skewness given by eq 12 was numerically inverted with the normalized topographical coefficients *h̅*_1_ to *h̅*_4_ equal to 5/6, 17/24, 49/80, and 43/80, respectively, that correspond to the case of spheroidal and spherical particles. In this way, the dependence of the layer coverage on the skewness was obtained, which is plotted in Figure 3 as a solid line.

As can be seen, the coverage monotonically decreases with the layer skewness, and this relationship was adequately interpolated by the fitting function
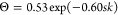
17shown as the dashed line in Figure 5. Thus, for *sk* = 0, the predicted layer coverage equals to 0.53 that represents the largest one occurring in the experimental measurements of nanoparticle deposition kinetics.^89^ Therefore, eq 17 can be used for the real-time measurements of particle or protein adsorption kinetics applying, for example, the high-speed AFM imaging technique if an appropriate software yielding the layer skewness for discrete time intervals was available.

### Modeling Results — Comparison with Experiments

The applicability of the above theoretical results was determined by performing computer modeling, where the extended random sequential adsorption approach (referred to as soft-RSA) was applied for the creation of particle layers (Supporting Information). The number of particles in the layer generated in a single modeling run was typically equal to 2 × 10^3^. Then, a rectangular net with a regulated distance between mesh points *d*_pix_ was superimposed and the topographical parameters for the particle layers were calculated as
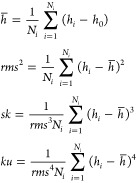
18where *N*_*i*_ is the number of mesh points (pixels) corresponding to the net size.

A sufficiently large number of independent runs were performed in order to attain a precision of these calculations of 0.001.

Calculations were carried out for a discrete set of particle coverages varying from 0.025 to 0.547, pertinent to the RSA jamming limit for spherical particles.^81^ The topographical parameters were additionally calculated for the regular particle monolayer characterized by a maximum coverage of 0.785 and the densely packed hexagonal monolayer characterized by a coverage of 0.907. The modeling was performed for the various pixel to particle size ratio *d*_pix_/*d_p_* equal to 0.1, 0.2, 0.5, and 1.

**Figure 4:**
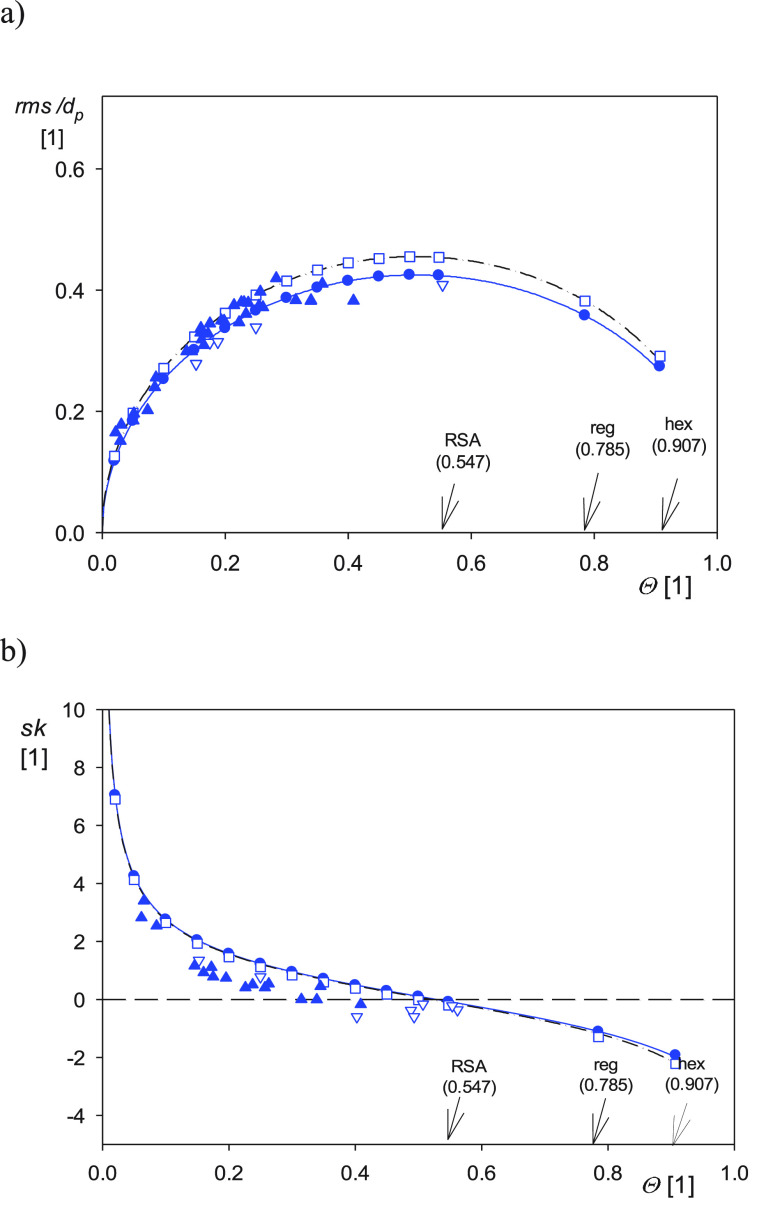
(a) Dependence of the normalized *rms* on the spherical particle layer coverage Θ. (b) Dependence of the skewness *sk* on the spherical particle layer coverage Θ. Unfilled squares denote the results derived from MC-RSA computer experiments for the pixel to particle size ratio equal to unity. Full circles denote the computer experiments for the pixel to particle size ratio 0.5 and 0.2. Full triangles denote the experimental AFM data derived for polymer particles on mica. Inverted triangles denote the experimental data for polymer particles on the silica sensor. The dashed/dotted line shows the interpolation of the theoretical data for the pixel to particle size ratio equal to unity, and the solid line shows the analytical results derived from eq 16.

**Figure 5:**
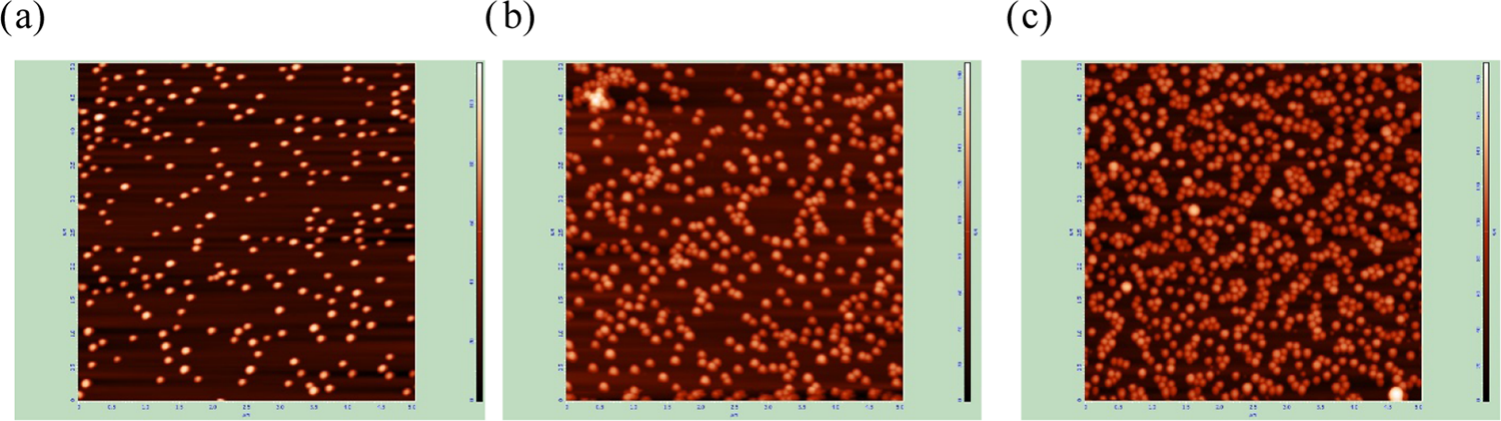
AFM micrographs of the A100 amidine particle layers on mica, scan size 5 × 5. The dimensionless particle coverage: (a) Θ = 0.074, (b) Θ = 0.14, and (c) Θ = 0.26.

In Figure 4a, the dependence of the normalized *rms*/*d_p_* derived from the modeling on the spherical particle layer coverage is presented for the pixel to particle size ratio equal to unity and 0.2. In the latter case, the numerical results coincided with the analytical ones calculated from eq 16. Also for *d*_pix_/*d_p_=* 0.5, the numerical results agree with the analytical data (to within 1% precision) and were not shown. Noticeable differences between the numerical and analytical results only appeared for *d*_pix_/*d_p_=* 1. In this case, the normalized *rms*/*d_p_* was larger for the entire coverage range (see the upper line in Figure 5a). Thus, the maximum value of *rms*/*d_p_* was equal to 0.455 compared to the analytical value of 0.425, which amounts to a 7% difference.

The results pertinent to the particle layer skewness are presented in Figure 4b. It can be seen that the numerical and analytical results agree with each other both for *d*_pix_/*d_p_=* 0.2 and 1, with the relative deviation not exceeding 1% for the coverage below 0.8.

These results have practical significance, indicating that the error stemming from the discretization of the particle layer in AFM measurements can be minimized if the pixel to particle size ratio *d*_pix_/*d_p_* is kept below 0.5.

The theoretical data shown in Figures 5a,5b were compared with the experimental measurements performed for positively charged amidine particles (zeta potential at pH 5.6 and 0.01 M NaCl equal to 60–70 mV) of the DLS size equal to 100 ± 5 and 120 ± 5 nm, which were deposited under diffusion on bare mica. Measurements were also performed for amidine particles of the sizes 70 ± 5 nm, 140 ± 5, and 350 ± 20 nm and albumin-covered polystyrene particles of the size 120 ± 5 nm,^90^ which were deposited under flow on the silica sensor in the QCM cell. The particles were imaged by AFM under ambient air conditions with the typical scan size of 5 × 5 or 2 × 2 μm for the smaller particles. The A100 particle layers deposited on mica under diffusion-controlled conditions (pH 5.6 and 10 mM NaCl concentration) are shown in Figure 5. For these scan areas and particle sizes, the pixel to particle size ratio *d*_pix_/*d_p_* was about 0.2. Considering the above theoretical results, such a small *d*_pix_/*d_p_* ratio ensures an adequate precision of the measurements. Additionally, to decrease the error stemming from the thermal drift, the particle coverage was calculated using the DLS sizes, considering the average number of particles per scan area. The topographical parameters were calculated from AFM scans using eq 18.

These experimental data obtained for various particle sizes are compared with the computer modeling data, as shown in Figures 4a,4b. As can be seen in the former figure, the experimental values of the normalized *rms* agree with the theoretical predictions for the broad range of the coverage attaining the RSA limit of 0.547. However, it should be mentioned that the relative precision of these measurements was about ±5%, mainly due to the error in the particle size determination. Additionally, as shown in the Supporting Information, for the layer coverage above 0.3, a systematic error may appear due to the AFM tip convolution effects. However, as shown in ref (75) for the particle to tip size ratio of 5 (corresponding to our experimental conditions), the relative *rms* decreased by 2–5% for two different types of rough surfaces.

In Figure 4a, the particle layer skewness derived from the AFM measurements is compared with the theoretical predictions. As can be seen, the experimental data well reflect the general trend, confirming a monotonic decrease of the skewness with the particle coverage. However, the experimental determination of the skewness is charged with a more significant error compared to the rms because the third-order moment is involved in its calculation (see eq 18). Therefore, a more precise estimation of the validity of the theoretical model can be achieved for AFM tips with a small radius of curvature and cone angle.

## Conclusions

A theoretical approach was formulated, enabling quantitative characteristics of particle layer topography. Analytical results were derived introducing the absolute coverage for particles of various shapes such as cylinders (rods), disks, ellipsoids, spheroids, spheres, hemispheres, etc.

It was shown that the *rms*, which was proportional to the dimension perpendicular to the surface, exhibited a maximum for all particle shapes at a coverage of about 0.5. In consequence, this parameter alone was not sufficient to unequivocally characterize particle layer topography. In contrast, the layer skewness was a monotonic function of the coverage, remaining independent of the particle size. It was also predicted that the differences in the skewness among various particles were practically negligible for the coverage below 0.5. Because of this property, the skewness can be used for a facile determination of the particle coverage, even if their shape and size are not known.

The applicability of the analytical results was confirmed by computer modeling performed according to the Monte Carlo random sequential adsorption (RSA) approach. The role of the discretization pertinent to the atomic force microscopy (AFM) measurements was evaluated. It was shown that for the pixel to particle size ratio *d*_pix_/*d_p_* of 0.5, the results derived from the modeling agreed within 1% with the analytical predictions, whereas for *d*_pix_/*d_p_* = 0.2, the difference decreased to 0.1%. These calculations have practical significance indicating that the systematic error stemming from the discretization of the particle layer in AFM measurements can be minimized if *d*_pix_/*d_p_* < 0.5.

Comparison with the experimental data acquired for polymer nanoparticle layers on mica and silica (QCM sensors) confirmed the trends predicted for the *rms* and the skewness, which exhibited a monotonic decrease with the coverage.

Therefore, results acquired in this work can be used for a topographical characteristization of the surface assemblies of carbon nanotubes, silica particles, vesicles, macroions, DNA chains, proteins, viruses, and bacteria. One can also expect that the results can serve as reference data for the interpretation of the rms and other topographical parameters of protein and nanoparticle layers on curved interfaces, which are difficult to experimentally acquire.

## Supporting Information Available

Calculations of the topographical parameters for particle-covered surfaces, modeling of particle layers - the Monte-Carlo RSA Approach and AFM measurements (PDF)

## References

[ref1] Pradeep T.; Anshup Noble metal nanoparticles for water purification: A critical review. Thin Solid Films 2009, 517, 6441–6478. 10.1016/j.tsf.2009.03.195.

[ref2] Puri N.; Gupta A.; Mishra A. Recent advances on nano-adsorbents and nanomembranes for the remediation of water. J. Clean Prod. 2021, 322, 129051 10.1016/j.jclepro.2021.129051.

[ref3] Yang S.; Pelton R.; Raegen A.; Montgomery M.; Dalnoki-Veress K. Nanoparticle flotation collectors: mechanisms behind a new technology. Langmuir 2011, 27, 10438–10446. 10.1021/la2016534. 21790133

[ref4] Nasirimoghaddam S.; Mohebbi A.; Karimi M.; Reza Yarahmadi M. Assessment of pH-responsive nanoparticles performance on laboratory column flotation cell applying a real ore feed. Int. J. Min Sci. Technol. 2020, 30, 197–205. 10.1016/j.ijmst.2020.01.001.

[ref5] Maenosono S.; Okubo T.; Yamaguchi Y. Overview of nanoparticle array formation by wet coating. J. Nanopart. Res. 2003, 5, 5–15. 10.1023/a:1024418931756.

[ref6] Altay B. N.; Klass C.; Chen T.; Fleck A.; Aydemir C.; Karademir A.; Fleming P. D. The effect of green biobased binder on structural, mechanical, liquid absorption and wetting properties of coated papers. Clean Eng. Technol. 2021, 5, 100274 10.1016/j.clet.2021.100274.

[ref7] Chiolerio A.; Maccioni G.; Martino P.; Cotto M.; Pandolfi P.; Rivolo P.; Ferrero S.; Scaltrito L. Inkjet printing and low power laser annealing of silver nanoparticle traces for the realization of low resistivity lines for flexible electronics. Microelectron. Eng. 2011, 88, 2481–2483. 10.1016/j.mee.2010.12.099.

[ref8] Wu Y.; Liu P.; Wigglesworth T. Development of silver nanoparticle ink for printed electronics. J. Microelectron Electron Packag. 2013, 10, 49–53. 10.4071/imaps.365.

[ref9] Bouafia A.; Laouini S. E.; Ahmed A. S. A.; Soldatov A. V.; Algarni H.; Feng Chong K.; Ali G. A. M. The recent progress on silver nanoparticles: Synthesis and electronic applications. Nanomaterials 2021, 11, 2318 10.3390/nano11092318. 34578634 PMC8467496

[ref10] McGrath F.; Qian J.; Gwynne K.; Kumah C.; Daly D.; Hrelescu C.; Zhang X.; O’Carroll D. M.; Bradley A. L. Structural, optical, and electrical properties of silver gratings prepared by nanoimprint lithography of nanoparticle ink. Appl. Surf. Sci. 2021, 537, 147892 10.1016/j.apsusc.2020.147892.

[ref11] Zhang X.; Xu S.; Jiang S.; Wang J.; Wei J.; Xu S.; Gao S.; Liu H.; Qiu H.; Li Z.; Liu H.; Li Z.; Li H. Growth graphene on silver–copper nanoparticles by chemical vapor deposition for high-performance surface-enhanced Raman scattering. Appl. Surf. Sci. 2015, 353, 63–70. 10.1016/j.apsusc.2015.06.084.

[ref12] Oćwieja M.; Adamczyk Z.; Morga M.; Kubiak K. Silver particle monolayers - formation, stability, applications. Adv. Colloid Interface Sci. 2015, 222, 530–563. 10.1016/j.cis.2014.07.001. 25169969

[ref13] Barbasz A.; Czyżowska A.; Piergies N.; Oćwieja M. Design cytotoxicity: The effect of silver nanoparticles stabilized by selected antioxidants on melanoma cells. J. Appl. Toxicol. 2022, 42, 570–587. 10.1002/jat.4240. 34558088

[ref14] Jiang Z.-J.; Liu C.-Y.; Sun L.-W. Catalytic properties of silver nanoparticles supported on silica spheres. J. Phys. Chem. B 2005, 109, 1730–1735. 10.1021/jp046032g. 16851151

[ref15] Gangula A.; Podila R.; Ramakrishna M.; Karanam L.; Janardhana C.; Rao A. M. Catalytic reduction of 4-nitrophenol using biogenic gold and silver nanoparticles derived from Breynia rhamnoides. Langmuir. 2011, 27, 15268–15274. 10.1021/la2034559. 22026721

[ref16] Liang Y. Q.; Cui Z. D.; Zhu S. L.; Liu Y.; Yang X. J. Silver nanoparticles supported on TiO_2_ nanotubes as active catalysts for ethanol oxidation. J. Catal. 2011, 278, 276–287. 10.1016/j.jcat.2010.12.011.

[ref17] Kulthong K.; Srisung S.; Boonpavanitchakul K.; Kangwansupamonkon W.; Maniratanachote R. Determination of silver nanoparticle release from antibacterial fabrics into artificial sweat. Part Fibre Toxicol. 2010, 7, 8 10.1186/1743-8977-7-8. 20359338 PMC2861638

[ref18] Tang B.; Zhang M.; Hou X.; Li J.; Sun L.; Wang X. Coloration of cotton fibers with anisotropic silver nanoparticles. Ind. Eng. Chem. Res. 2012, 51, 12807–12813. 10.1021/ie3015704.

[ref19] Cai H.; Xu C.; He P.; Fang Y. Colloid Au-enhanced DNA immobilization for the electrochemical detection of sequence-specific DNA. J. Electroanal. Chem. 2001, 510, 78–85. 10.1016/S0022-0728(01)00548-4.

[ref20] Zhong J.; Qi Z.; Dai H.; Fan C.; Li G.; Matsuda N. Sensing phenothiazine drugs at a gold electrode co-modified with DNA and gold nanoparticles. Anal. Sci. 2003, 19, 653–657. 10.2116/analsci.19.653. 12769359

[ref21] Chen Q.; Tang W.; Wang D.; Wu X.; Li N.; Liu F. Amplified QCM-D biosensor for protein based on aptamer-functionalized gold nanoparticles. Biosens. Bioelectron. 2010, 26, 575–579. 10.1016/j.bios.2010.07.034. 20692147

[ref22] Mazeiko V.; Kausaite-Minkstimiene A.; Ramanaviciene A.; Balevicius Z.; Ramanavicius A. Gold nanoparticle and conducting polymer-polyaniline-based nanocomposites for glucose biosensor design. Sens. Actuators, B 2013, 189, 187–193. 10.1016/j.snb.2013.03.140.

[ref23] German N.; Ramanavicius A.; Ramanaviciene A. Electrochemical deposition of gold nanoparticles on graphite rod for glucose biosensing. Sens. Actuators, B 2014, 203, 25–34. 10.1016/j.snb.2014.06.021.

[ref24] Fan M.; Brolo A. G. Self-assembled Au nanoparticles as substrates for surface-enhanced vibrational spectroscopy: Optimization and electrochemical stability. ChemPhysChem. 2008, 9, 1899–1907. 10.1002/cphc.200800099. 18704901

[ref25] Fan M.; Andrade G. F. S.; Brolo A. G. A review on the fabrication of substrates for surface enhanced Raman spectroscopy and their applications in analytical chemistry. Anal. Chim. Acta 2011, 693, 7–25. 10.1016/j.aca.2011.03.002. 21504806

[ref26] Ramalingam V. Multifunctionality of gold nanoparticles: Plausible and convincing properties. Adv. Colloid Interface Sci. 2019, 271, 101989 10.1016/j.cis.2019.101989. 31330396

[ref27] Kim N. H.; Baek T. J.; Park H. G.; Seong G. H. Highly sensitive biomolecule detection on a quartz crystal microbalance using gold nanoparticles as signal amplification probes. Anal. Sci. 2007, 23, 177–181. 10.2116/analsci.23.177. 17297229

[ref28] Ishida T.; Kuroda K.; Kinoshita N.; Minagawa W.; Haruta M. Direct deposition of gold nanoparticles onto polymer beads and glucose oxidation with H_2_O_2_. J. Colloid Interface Sci. 2008, 323, 105–111. 10.1016/j.jcis.2008.02.046. 18387617

[ref29] Das S.; Asefa T. Core–shell–shell microsphere catalysts containing Au nanoparticles for styrene epoxidation. Top Catal. 2012, 55, 587–594. 10.1007/s11244-012-9835-x.

[ref30] Cheval N.; Gindy N.; Flowkes C.; Fahmi A. Polyamide 66 microspheres metallised with in situ synthesised gold nanoparticles for a catalytic application. Nanoscale Res. Lett. 2012, 7, 182 10.1186/1556-276X-7-182. 22401661 PMC3323437

[ref31] Schauermann S.; Nilius N.; Shaikhutdinov S.; Freund H.-J. Nanoparticles for heterogeneous catalysis: new mechanistic insights. Acc. Chem. Res. 2013, 46, 1673–1681. 10.1021/ar300225s. 23252628

[ref32] Shi L.; Zhu L.; Guo J.; Zhang L.; Shi Y.; Zhang Y.; Hou K.; Zheng Y.; Zhu Y.; Lv J.; Liu S.; Tang Z. Self-assembly of chiral gold clusters into crystalline nanocubes of exceptional optical activity. Angew. Chem., Int. Ed. 2017, 56, 15397–15401. 10.1002/anie.201709827. 29057591

[ref33] Lotito V.; Zambelli T. Pattern detection in colloidal assembly: A mosaic of analysis techniques. Adv. Colloid Interface Sci. 2020, 284, 102252 10.1016/j.cis.2020.102252. 32971396

[ref34] Park S. I.; Song H.-M. Synthesis of prolate-shaped Au nanoparticles and Au nanoprisms and study of catalytic reduction reactions of 4-nitrophenol. ACS Omega 2019, 4, 7874–7883. 10.1021/acsomega.9b00647. 31459874 PMC6647965

[ref35] Pol V. G.; Calderon-Moreno J. M.; Chupas P. J.; Winans R. E.; Thiyagarajan P. Synthesis of monodispersed prolate spheroid shaped paramagnetic carbon. Carbon 2009, 47, 1050–1055. 10.1016/j.carbon.2008.12.028.

[ref36] Wu L.; Gao B.; Tian Y.; Muñoz-Carpena R.; Zigler K. J. DLVO Interactions of carbon nanotubes with isotropic planar surfaces. Langmuir 2013, 29, 3976–3988. 10.1021/la3048328. 23442014

[ref37] Gomez-Flores A.; Bradford S. A.; Wu L.; Kim H. Interaction energies for hollow and solid cylinders: Role of aspect ratio and particle orientation. Colloids Surf., A 2019, 580, 123781 10.1016/j.colsurfa.2019.123781.

[ref38] van Kats C. M.; Johnson P. M.; van den Meerakker J. E. A. M.; van Blaaderen A. Synthesis of monodisperse high-aspect-ratio colloidal silicon and silica rods. Langmuir 2004, 20, 11201–11207. 10.1021/la048817j. 15568876

[ref39] Kuijk A.; van Blaaderen A.; Imhof A. Synthesis of monodisperse, rodlike silica colloids with tunable aspect ratio. J. Am. Chem. Soc. 2011, 133, 2346–2349. 10.1021/ja109524h. 21250633

[ref40] Kuijk A.; Imhof A.; Verkuijlen M. H. W.; Besseling T. H.; van Eck E. R. H.; van Blaaderen A. Colloidal silica rods: Material properties and fluorescent labeling. Part. Part. Syst. Charact. 2014, 31, 706–713. 10.1002/ppsc.201300329.

[ref41] Bakker H. E.; Besseling T. H.; Wijnhoven J. E. G. J.; Helfferich P. H.; van Blaaderen A.; Imhof A. Microelectrophoresis of silica rods using confocal microscopy. Langmuir 2017, 33, 881–890. 10.1021/acs.langmuir.6b03863. 28045541 PMC5348103

[ref42] Ho C. C.; Ottewill R. H.; Keller A.; Odell J. A. Monodisperse ellipsoidal polystyrene particles: Preparation and characterization. Colloids Polym. Sci. 1993, 271, 469–479. 10.1007/bf00657391.

[ref43] Champion J. A.; Katare Y. K.; Mitragotri S. Making Polymeric micro- and nanoparticles of complex shapes. Proc. Natl. Acad. Sci. U.S.A. 2007, 104, 11901–11904. 10.1073/pnas.0705326104. 17620615 PMC1924596

[ref44] Gadzinowski M.; Mickiewicz D.; Basińska T. Spherical versus prolate spheroidal particles in biosciences: Does the shape make a difference?. Polym. Adv. Technol. 2021, 32, 3867–3876. 10.1002/pat.5413.

[ref45] Tirado M. M.; Martínez C. L.; de la Torre J. G. Comparison of theories for the translational and rotational diffusion coefficients of rod-like macromolecules. Application to short DNA fragments. J. Chem. Phys. 1984, 81, 2047–2052. 10.1063/1.447827.

[ref46] Allison S. A.; Mazur S. Modeling the free solution electrophoretic mobility of short DNA fragments. Biopolymers 1998, 46, 359–373. 10.1002/(sici)1097-0282(199811)46:63.0.co;2-#.

[ref47] Liu L.; Guo Z.; Huang Z.; Zhuang J.; Yang W. Size-selective separation of DNA fragments by using lysine-functionalized silica particles. Sci. Rep. 2016, 6, 22029 10.1038/srep22029. 26911527 PMC4766563

[ref48] Cieśla M.; Adamczyk Z.; Barbasz J.; Wasilewska M. Mechanisms of fibrinogen adsorption at solid substrates at lower pH. Langmuir 2013, 29, 7005–7016. 10.1021/la4012789. 23621148

[ref49] Sha J.; Si W.; Xu B.; Zhang S.; Li K.; Lin K.; Shi H.; Chen Y. Identification of spherical and nonspherical proteins by a solid-state nanopore. Anal. Chem. 2018, 90, 13826–13831. 10.1021/acs.analchem.8b04136. 30406650

[ref50] Dogic Z.; Fraden S. Ordered phases of filamentous viruses. Curr. Opin. Colloid Interface Sci. 2006, 11, 47–55. 10.1016/j.cocis.2005.10.004.

[ref51] Buitenhuis J. Electrophoresis of Fd-virus particles: experiments and an analysis of the effect of finite rod lengths. Langmuir 2012, 28, 13354–13363. 10.1021/la302245x. 22958165

[ref52] Young K. D. The selective value of bacterial shape. Microbiol. Mol. Biol. Rev. 2006, 70, 660–703. 10.1128/MMBR.00001-06. 16959965 PMC1594593

[ref53] Koch A. L. Shapes that *Escherichia Coli* cells can achieve, as a paradigm for other bacteria. Crit. Rev. Microbiol. 2005, 31, 183–190. 10.1080/10408410590928504. 16170908

[ref54] Kleimann J.; Lecoultre G.; Papastavrou G.; Jeanneret S.; Galletto P.; Koper G. J.; Borkovec M. Deposition of nanosized latex particles onto silica and cellulose surfaces studied by optical reflectometry. J. Colloid Interface Sci. 2006, 303, 460–471. 10.1016/j.jcis.2006.08.006. 16978638

[ref55] Toccafondi C.; Prato M.; Maidecchi G.; Penco A.; Bisio F.; Cavalleri O.; Canepa M. Optical properties of Yeast Cytochrome c monolayer on gold: An in situ spectroscopic ellipsometry investigation. J. Colloid Interface Sci. 2011, 364, 125–132. 10.1016/j.jcis.2011.07.097. 21920531

[ref56] Höök F.; Vörös J.; Rodahl M.; Kurrat R.; Böni P.; Ramsden J. J.; Textor M.; Spencer N. D.; Tengvall P.; Gold J.; Kasemo B. A comparative study of protein adsorption on titanium oxide surfaces using in situ ellipsometry, optical waveguide lightmode spectroscopy, and quartz crystal microbalance/dissipation. Colloids Surf., B 2002, 24, 155–170. 10.1016/S0927-7765(01)00236-3.

[ref57] Sander M.; Madliger M.; Schwarzenbach R. P. Adsorption of transgenic insecticidal Cry1Ab protein to SiO_2_. 1. Forces driving adsorption. Environ. Sci. Technol. 2010, 44, 8870–8876. 10.1021/es103008s. 21033745

[ref58] Wasilewska M.; Adamczyk Z.; Sadowska M.; Boulmedais F.; Cieśla M. Mechanisms of fibrinogen adsorption on silica sensors at various pHs: experiments and theoretical modeling. Langmuir 2019, 35, 11275–11284. 10.1021/acs.langmuir.9b01341. 31394033

[ref59] Reimhult E.; Larsson C.; Kasemo B.; Höök F. Simultaneous surface plasmon resonance and quartz crystal microbalance with dissipation monitoring measurements of biomolecular adsorption events involving structural transformations and variations in coupled water. Anal. Chem. 2004, 76, 7211–7220. 10.1021/ac0492970. 15595862

[ref60] Olsson A. L. J.; Quevedo I. R.; He D.; Basnet M.; Tufenkji N. Using the quartz crystal microbalance with dissipation monitoring to evaluate the size of nanoparticles deposited on surfaces. ACS Nano 2013, 7, 7833–7843. 10.1021/nn402758w. 23964846

[ref61] Chen Q.; Xu S.; Liu Q.; Masliyah J.; Xu Z. QCM-D study of nanoparticle interactions. Adv. Colloid Interface Sci. 2016, 233, 94–114. 10.1016/j.cis.2015.10.004. 26546115

[ref62] Tarnapolsky A.; Freger V. Modeling QCM-D response to deposition and attachment of microparticles and living cells. Anal. Chem. 2018, 90, 13960–13968. 10.1021/acs.analchem.8b03411. 30295025

[ref63] Adamczyk Z.; Sadowska M.; Żeliszewska P. Applicability of QCM-D for quantitative measurements of nano- and microparticle deposition kinetics: theoretical modeling and experiments. Anal. Chem. 2020, 92 (22), 15087–15095. 10.1021/acs.analchem.0c03115. 32957771 PMC7675609

[ref64] Gopalakrishna S.; Langhoff A.; Brenner G.; Johansmann D. Soft viscoelastic particles in contact with a quartz crystal microbalance (QCM): A frequency-domain lattice Boltzmann simulation. Anal. Chem. 2021, 93, 10229–10235. 10.1021/acs.analchem.1c01612. 34270892

[ref65] Adamczyk Z.; Sadlej K.; Wajnryb E.; Nattich M.; Ekiel-Jeżewska M.; Bławzdziewicz J. Streaming potential studies of colloid, polyelectrolytes and protein deposition. Adv. Colloid Interface Sci. 2010, 153, 1–29. 10.1016/j.cis.2009.09.004. 19926067

[ref66] Cross W. M.; Ma S.; Winter R. M.; Kellar J. J. FT-IR and SEM study of colloidal particle deposition. Colloids Surf., A 1999, 154, 115–125. 10.1016/s0927-7757(98)00914-5.

[ref67] Klapetek P.; Valtr M.; Nečas D.; Salyk O.; Dzik P. Atomic force microscopy analysis of nanoparticles in non-ideal conditions. Nanoscale Res. Lett. 2011, 6, 514 10.1186/1556-276X-6-514. 21878120 PMC3212053

[ref68] Dallaeva D.; Ţălu S.; Stach S.; Škarvada P.; Tománek P.; Grmela L. AFM imaging and fractal analysis of surface roughness of AlN epilayers on sapphire substrates. Appl. Surf. Sci. 2014, 312, 81–86. 10.1016/j.apsusc.2014.05.086.

[ref69] Morga M.; Nattich-Rak M.; Oćwieja M.; Adamczyk Z. Gold substrates of controlled roughness and electrokinetic properties formed by nanoparticle deposition. Phys. Chem. Chem. Phys. 2019, 21, 6535–6543. 10.1039/C9CP00440H. 30843905

[ref70] Ando T.; Kodera N.; Uchihashi T.; Miyagi A.; Nakakita R.; Yamashita H.; Matada K. High-speed atomic force microscopy for capturing dynamic behavior of protein molecules at work. e-J. Surf. Sci. Nanotechnol. 2005, 3, 384–392. 10.1380/ejssnt.2005.384.

[ref71] Ando T. High-speed atomic force microscopy. Curr. Opin. Chem. Biol. 2019, 51, 105–112. 10.1016/j.cbpa.2019.05.010. 31254806

[ref72] Xin Y.; Grundmeier G.; Keller A. Adsorption of SARS-CoV-2 spike protein S1 at oxide surfaces studied by high-speed atomic force microscopy. Adv. Nanobiomed Res. 2021, 1, 2000024 10.1002/anbr.202170023. 33615316 PMC7883093

[ref73] Morga M.; Nattich-Rak M.; Adamczyk Z.; Mickiewicz D.; Gadzinowski M.; Basinska T. Mechanisms of anisotropic particle deposition: prolate spheroid layers on mica. J. Phys.Chem. C 2022, 126, 18550–18559. 10.1021/acs.jpcc.2c06028.

[ref74] Shen J.; Zhang D.; Zhang F.-H.; Gan Y. AFM tip-sample convolution effects for cylinder protrusions. Author links open overlay panel. Appl. Surf. Sci. 2017, 422, 482–491. 10.1016/j.apsusc.2017.06.053.

[ref75] Westra K. L.; Thomson D. J. Effect of tip shape on surface roughness measurements from atomic force microscopy images of thin films. J. Vac. Sci. Technol. 1995, 13, 344–349. 10.1116/1.587943.

[ref76] Batys P.; Weroński P. Modeling of LbL multilayers with controlled thickness, roughness and specific surface area. J. Chem. Phys. 2012, 137, 214706 10.1063/1.4769390. 23231255

[ref77] Hinrichsen E. L.; Feder J.; Jøssang T. Geometry of random sequential adsorption. J. Stat. Phys. 1986, 44, 793–827. 10.1007/BF01011908.

[ref78] Viot P.; Tarjus G.; Ricci S. M.; Talbot J. Random sequential adsorption of anisotropic particles. I. Jamming limit and asymptotic behavior. J. Chem. Phys. 1992, 97, 5212–5218. 10.1063/1.463820.

[ref79] Evans J. W. Random and cooperative sequential adsorption. Rev. Mod. Phys. 1993, 65, 1281–1329. 10.1103/RevModPhys.65.1281.

[ref80] Talbot J.; Tarjus G.; van Tassel P. R.; Viot P. From car parking to protein adsorption: an overview of sequential adsorption processes. Colloids Surf., A 2000, 165, 287–324. 10.1016/S0927-7757(99)00409-4.

[ref81] Adamczyk Z. Particles at interfaces: Interactions, Deposition, Structure; Elsevier: London, 2017.

[ref82] Oshima H.; Furusawa K. Electrical phenomena at Interfaces: Fundamentals, Measurements, and Applications; Marcel Dekker, Inc.: New York, 1998.

[ref83] Zembala M.; Adamczyk Z. Measurements of streaming potential for mica covered by colloid particles. Langmuir 2000, 16, 1593–1601. 10.1021/la9905970. 20364856

[ref84] Daikhin L.; Gileadi E.; Katz G.; Tsionsky Y.; Urbakh M.; Zagidulin D. Influence of roughness on the admittance of the quartz crystal microbalance immersed in liquids. Anal. Chem. 2002, 74, 554–561. 10.1021/ac0107610. 11838676

[ref85] McHale G.; Newton M. I. Surface roughness and interfacial slip boundary condition for quartz crystal microbalances. J. Appl. Phys. 2004, 95, 373–380. 10.1063/1.1630373.

[ref86] Rechendorff K.; Hovgaard M. B.; Foss M.; Besenbacher F. Influence of surface roughness on quartz crystal microbalance measurements in liquids. J. Appl. Phys. 2007, 101, 114502 10.1063/1.2735399.

[ref87] Pelliccione M.; Karabacak T.; Gaire C.; Wang G.-C.; Lu T.-M. Mound formation in surface growth under shadowing. Phys. Rev. B 2006, 74, 125420 10.1103/PhysRevB.74.125420. 16712101

[ref88] Peter B.; Kurunczi S.; Patko D.; Lagzi I.; Kowalczyk B.; Rácz Z.; Grzybowski B. A.; Horvath R. Label-free in situ optical monitoring of the adsorption of oppositely charged metal nanoparticles. Langmuir. 2014, 30 (44), 13478–13482. 10.1021/la5029405. 25361404

[ref89] Adamczyk Z.; Morga M.; Nattich-Rak M.; Sadowska M. Nanoparticle and bioparticle deposition kinetics. Adv. Colloid Interface Sci. 2022, 302, 102630 10.1016/j.cis.2022.102630. 35313169

[ref90] Nattich-Rak M.; Sadowska M.; Motyczyńska M.; Adamczyk Z. Mimicking pseudo-virion interactions with abiotic surfaces: Deposition of polymer nanoparticles with albumin corona. Biomolecules 2022, 12, 1658 10.3390/biom12111658. 36359008 PMC9687657

