# Supplementary material for: Quantifying Nanoparticle Layer Topography: Theoretical Modeling and Atomic Force Microscopy Investigations
Source: Langmuir. 2023 Oct 12;39(42):15067–77. doi: 10.1021/acs.langmuir.3c02024 (PMC10601541; doi:10.1021/acs.langmuir.3c02024)
Supplement: Supplementary file 1 — la3c02024_si_001.pdf [file la3c02024_si_001.pdf]

Supporting Information

Quantifying Nanoparticle Layer Topography:

Theoretical Modeling and Atomic Force Microscopy Investigations

Zbigniew Adamczyk, Marta Sadowska*, Małgorzata Nattich-Rak*

Jerzy Haber Institute of Catalysis and Surface Chemistry, Polish Academy of Sciences, Niezapominajek 8, 30 - 239 Krakow, Poland

*Corresponding authors: e-mail: zbigniew.adamczyk@ikifp.edu.pl
marta.sadowska@ikifp.edu.pl

Table of contents:

1. Calculations of the topographical parameters for particle covered surfaces
2. Modeling of Particle Layers - the Monte-Carlo RSA Approach
3. AFM Measurements – Determination of the Topographical Parameters
4. APPENDIX A (Supplement to Section 1)

1. Calculations of the topographical parameters for particle covered surfaces

The constitutive expression for the average height $\bar{h}$ of a rough surface is the following

$$\bar{h} = \frac{1}{S}\int_s \left(h\left(\mathbf{r}_s\right) - h_0\right)\cdot d\mathbf{r}_s = h_1 - h_0 \tag{S1}$$

where S is the projection area of the surface, $h(\mathbf{r}_s)$ is the local height of the surface profile measured relatively to a reference planar surface located at h_0, $\mathbf{r}_s$ is the surface position vector and

$$h_1 = \frac{1}{S}\int_s h\left(\mathbf{r}_s\right)\cdot d\mathbf{r}_s \tag{S2}$$

It is evident from eq S1 that the definition of the average height is not unique because it depends on the position of the reference plane.

In contrast, all the central moments of the surface μ_q can be uniquely calculated from the general formula

$$\mu_q = \frac{1}{S}\int_s \left[h\left(\mathbf{r}_s\right) - \bar{h}\right]^q \cdot d\mathbf{r}_s \tag{S3}$$

where q = 2,3,4…

It should be noted that $\mu_q(h - h_0) = \mu_q(h)$, thus the moments are independent of the location of the reference plane.

Explicitly, the second, third and fourths moments can be expressed as

$$\mu_2 = \frac{1}{S}\int_s \left[h\left(\mathbf{r}_s\right) - \bar{h}\right]^2 \cdot d\mathbf{r}_s = h_2 - h_1^2$$

$$\mu_3 = \frac{1}{S}\int_s \left[h(\mathbf{r}_s) - \bar{h}\right]^3 \cdot d\mathbf{r}_s = h_3 - 3h_2h_1 + 2h_1^3 \tag{S4}$$

$$\mu_4 = \frac{1}{S}\int_s \left[h(\mathbf{r}_s) - \bar{h}\right]^4 \cdot d\mathbf{r}_s = h_4 - 4h_3h_1 + 6h_2h_1^2 - 3h_1^4$$

where h_1 is defined by eq S2 and the remaining surface integrals h_2 to h_4 are given by

$$h_2 = \frac{1}{S}\int_s h^2(\mathbf{r}_s) \cdot d\mathbf{r}_s$$

$$h_3 = \frac{1}{S}\int_s h^3(\mathbf{r}_s) \cdot d\mathbf{r}_s \tag{S5}$$

$$h_4 = \frac{1}{S}\int_s h^4(\mathbf{r}_s) \cdot d\mathbf{r}_s$$

Consequently, the basic topographical parameters such as the root mean square (*rms*), the skewness (*sk*) and the kurtosis (*ku*) of a rough surface are defined as follows

$$rms^2 = \mu_2 = h_2 - h_1^2$$

$$sk = \mu_3 / rms^3 = (h_3 - 3h_2h_1 + 2h_1^3)/(h_2 - h_1^2)^{3/2} \tag{S6}$$

$$ku = \mu_4 / rms^4 = (h_4 - 4h_3h_1 + 6h_2h_1^2 - 3h_1^4)/(h_2 - h_1^2)^2$$

If the roughness stems from the presence of surface features (particles) attached to a planar surface located at $h = 0$, one can express the topographical parameters in a useful analytical form. Let us, therefore, assume that there are N_p features of equal size and the same shape, whose characteristic cross-section area is S_p (see Figure S1).

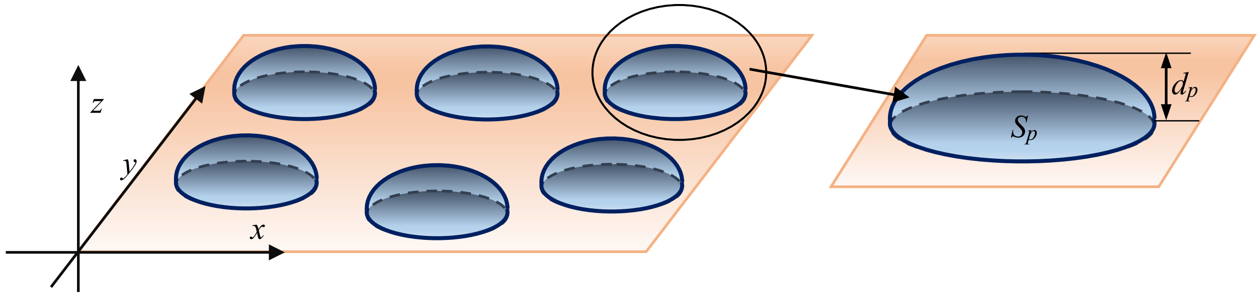

Figure S1. Schematic view of an interface covered by surface features (particles) of equal size with the characteristic dimension d_p.

Under such situation the overall surface integrals defined by eq S5 are reduced to the N_p equal integrals calculated over the surface area of S_p. In consequence the average height and other topographical parameters of the particle layer can be expressed as

$$\begin{aligned}
\bar{h} &= \left[\int_{s_p} h(\mathbf{r}_s) \cdot d\mathbf{r}_s \right] \frac{N_p S_p}{S} = h_{1p}\Theta \\
rms^2 &= \mu_2 = h_{2p}\Theta - h_{1p}\Theta^2 \\
sk &= \mu_3 / rms^3 = \left(h_{3p} - 3\, h_{2p} h_{1p}\Theta + 2\, h_{1p}^3 \Theta^2 \right) / \left[\Theta^{1/2} \left(h_{2p} - h_{1p}^2 \Theta \right)^{3/2} \right] \\
ku &= \mu_4 / rms^4 = \left(h_{4p} - 4\, h_{3p} h_{1p}\Theta + 6\, h_{2p} h_{1p}^2 \Theta^2 - 3\, h_{1p}^4 \Theta^3 \right) / \left[\Theta \left(h_{2p} - h_{1p}^2 \Theta \right)^2 \right]
\end{aligned} \tag{S7}$$

where

$$\begin{aligned}
h_{1p} &= \frac{1}{S_p} \int_{s_p} h(\mathbf{r}_s) \cdot d\mathbf{r}_s \\
h_{2p} &= \frac{1}{S_p} \int_{s_p} h^2(\mathbf{r}_s) \cdot d\mathbf{r}_s \\
h_{3p} &= \frac{1}{S_p} \int_{s_p} h^3(\mathbf{r}_s) \cdot d\mathbf{r}_s \\
h_{4p} &= \frac{1}{S_p} \int_{s_p} h^4(\mathbf{r}_s) \cdot d\mathbf{r}_s
\end{aligned} \tag{S8}$$

and

$$\Theta = N_p S_p / S \tag{S9}$$

is the absolute (thermodynamic) particle coverage.

It should be underlined that eq S7 is valid for arbitrary coverage and distribution of particles within the layer. It is also applicable for any particle shape given that the double integrals defined by eq S8 can be evaluated either analytically or by numerical techniques. Obviously, the analytical solution are of a special significance because they can be used for the formulation of

general laws governing topography of particle layers and for estimation of the precision of numerical solutions. In this respect, simple expression can be obtained for the category of particles characterized by a cross-section area independent of the coordinate perpendicular to the planar surface. This comprises cylinders of arbitrary, e.g., elliptic, cross-section area, disks, parallelepipeds (cubes), etc. For all these particles all the double integrals defined by eq S8 can be evaluated because $h(\mathbf{r}_s)$ is independent of the position vector. In consequence the and h_{1p} to h_{4p} coefficients become

$$
\begin{aligned}
h_{1p} &= d_p \\
h_{2p} &= d_p^2 \\
h_{3p} &= d_p^3 \\
h_{4p} &= d_p^4
\end{aligned}
\tag{S10}
$$

where d_p is the characteristic particle dimension perpendicular to the surface (see Figure S1).

Thus, according to eq S7 the average height, the root mean square (*rms*), the skewness (*sk*) and the kurtosis (*ku*) of the particle layer are given by

$$
\begin{aligned}
\bar{h} &= d_p \Theta \\
rms^2 &= d_p^2\, \Theta(1-\Theta) \\
sk &= \left(1-3\Theta+2\Theta^2\right)/\left[\Theta^{1/2}\left(1-\Theta\right)^{3/2}\right] \\
ku &= \left(1-4\Theta+6\Theta^2-3\Theta^3\right)/\left[\Theta\left(1-\Theta\right)^2\right]
\end{aligned}
\tag{S11}
$$

Eq S11 indicates that the average layer height and its *rms* scale up linearly with the particle dimension d_p, whereas the skewness and kurtosis are independent of the particle dimension.

The h_{1p} to h_{4p} coefficients were also derived for the particles in the form of elliptic caps, sphero-cylinders and cylinders aligned parallel to the interface, by evaluating the corresponding double-integrals in the Cartesian coordinate system (see Appendix A). For sake of convenience these coefficients were normalized as follows

$$\bar{h}_1 = h_{1p} / d_p$$
$$\bar{h}_2 = h_{2p} / d_p^2$$
$$\bar{h}_3 = h_{3p} / d_p^3 \qquad \text{(S12)}$$
$$\bar{h}_4 = h_{4p} / d_p^4$$

Accordingly, the topographical parameters of surfaces covered by such particle class are given by

$$\bar{h} = d_p \bar{h}_1 \Theta$$
$$rms^2 = d_p^2 \left(\bar{h}_2 \Theta - \bar{h}_1^2 \Theta^2 \right)$$
$$sk = \left(\bar{h}_3 - 3\bar{h}_2 \bar{h}_1 \Theta + 2\bar{h}_1^3 \Theta^2 \right) / \left[\Theta^{1/2} \left(\bar{h}_2 - \bar{h}_1^2 \Theta \right)^{3/2} \right] \qquad \text{(S13)}$$
$$ku = \left(\bar{h}_4 - 4\bar{h}_3 \bar{h}_1 \Theta + 6 \bar{h}_2 \bar{h}_1^2 \Theta^2 - 3 \bar{h}_1^4 \Theta^3 \right) / \left[\Theta \left(\bar{h}_2 - \bar{h}_1^2 \Theta \right)^2 \right]$$

The $\bar{h}_1$ - $\bar{h}_4$ coefficients are collected in Table 1 in the main manuscript where also the maximum values of the normalized rms/d_p are given a*s* well as the coverage where the maximum occurs.

As can be inferred from eq S13, for particle shapes, the average layer and the *rms* of the layers scale up linearly with the characteristic particle dimension d_p (perpendicular to the surface), whereas the skewness and kurtosis only depend on the coverage. The latter property has interesting practical repercussions enabling the determination of the particle layer coverage using the data acquired from experiments, e.g., AFM.

Moreover, using eq S13 one can formulate important limiting expression valid low coverage range where $\Theta << 1$, which assume the following form

$$\bar{h} = d_p \bar{h}_1 \Theta$$
$$rms = d_p \bar{h}_2^{1/2} \Theta^{1/2}$$
$$sk = (\bar{h}_3 / \bar{h}_2^{3/2}) \Theta^{-1/2} \qquad \text{(S14)}$$
$$ku = (\bar{h}_4 / \bar{h}_2^2) \Theta^{-1}$$

Thus, under this regime, the average layer height linearly increases with the particle coverage, the *rms* is proportional to the square root of the coverage and the skewness is proportional to $\Theta^{-1/2}$. This indicates that the sensitivity of the latter parameter to the particle coverage becomes exceptionally large in this limit increasing measurement sensitivity.

2. Modeling of Particle Layers - the Monte-Carlo RSA Approach

Computer experiments aimed at the determination of the above define topographical parameters were carried out according to the algorithm comprising the following steps:

(i) creation of the particle layer of a desired coverage applying a Monte-Carlo type random sequential adsorption (RSA) approach [1-7] for various physicochemical parameters related to experimental conditions,

(ii) quantitative characteristic of the layer structure in terms of the radial distribution function,

(iii) superimposition of a regular net over the particle layer characterized by the mesh size corresponding to the range of atomic force microscopy scans,

(iv) calculation of the topographical parameters for the discrete set of points resulting from the mesh point distribution.

For the creation of the particle layer, the extended RSA modeling (referred to as soft-RSA) was applied considering the electrostatic interactions of the incoming particle with all previously attached to the surface. The calculation algorithm was based on the following rules [1-3,5] a virtual molecule of a fixed size was generated whose position within the adsorption domain was selected at random; if the particle fulfilled defined adsorption criteria it was treated as firmly attached to the surface, thus its position was unchanged during the entire process; if the adsorption criteria were not fulfilled, a new adsorption attempt was made uncorrelated with previous ones. Two necessary deposition criteria corresponding to experimental measurements were adopted: no overlapping of the virtual particle with others attached to the surface and (ii) the availability of uncovered surface area on the substrate surface large enough to accommodate the virtual particle.

Because of the simplicity of governing rules, this RSA based algorithm enabled to generate large populations of deposited particles. In this way, the information about the deposition

kinetics, the maximum coverage, the blocking function and various correlation functions characterizing the distribution of particles in the layers can be extracted with a sufficient precision [7].

The net interaction energy of the virtual particle ϕ_v was calculated by summing the pair potentials within the interaction zone expressed using the linear superposition approach [6].

$$\phi_v = \sum_{i=1}^{N_i} \phi_{vi} \tag{S15}$$

where

$$\phi_{vi} = \frac{\phi_0}{r_{vi}} e^{-\kappa(r_{vi}-a_p)} \tag{S16}$$

and ϕ_{vi} is the interaction energy of the virtual particle with the i-th particle attached to the substrate, ϕ_0 is the pair energy on contact, r_{vi} is the distances between their centers, $a_p = d_p/2$ is the radius of the particle, $\kappa^{-1} = \left(\frac{\varepsilon kT}{2e^2 I}\right)^{1/2}$ is the electrical double-layer thickness, ε is the permittivity of the medium, k is the Boltzmann constant, T is the absolute temperature, e is the elementary charge and I is the ionic strength of the electrolyte solution.

Upon evaluating the interaction energy, the deposition probability density at a given point at the surface was calculated from the Boltzmann formula:

$$\rho_p = e^{-\phi_2/kT} \tag{S17}$$

The number of particles in the layer generated in a single modeling run N_p was typically equal to 2×10^3. In order to increase the precision of the modeling, a few independent runs were performed.

The absolute coverage of particles is define as before

$$\Theta = \frac{S_p N_p}{S} \tag{S18}$$

The particle distribution within the layer can be conveniently characterized in terms of the radial distribution function $g(r)$, referred also to as the pair distribution function). This function reflects the probability (averaged over the entire population) to find a particle pair separated by the distance r normalized to the uniform probability at large distances. If a sufficiently large particle population is available from the modeling this function can be calculated in a discrete form using the finite-difference equation [7]

$$g_i(r) = \frac{S_p}{\Theta} < N_{pi} / 2\pi r \Delta r > \tag{S19}$$

where N_{pi} is the number of particles within the ring $2\pi r \Delta r$ drawn around the central particle and < > means the ensemble average.

The topographical parameters for the particle layers generated in the modeling were calculated as

$$\begin{aligned}
\bar{h} &= \frac{1}{N_i}\sum_{i=1}^{N_i}\left(h_i - h_0\right) \\
rms^2 &= \frac{1}{N_i}\sum_{i=1}^{N_i}\left(h_i - \bar{h}\right)^2 \\
sk &= \frac{1}{rms^3 N_i}\sum_{i=1}^{N_i}\left(h_i - \bar{h}\right)^3 \\
ku &= \frac{1}{rms^4 N_i}\sum_{i=1}^{N_i}\left(h_i - \bar{h}\right)^4
\end{aligned} \tag{S20}$$

where N_i is the number of mesh points (pixels) corresponding to the net superimposed on the particle layer.

3. AFM characteristics of particle layers

It should be mentioned that all the above defined topographical parameters correspond to the situation where the entire interface area uncovered by particles is accessible to the scanning probe tip. However, in the case of AFM measurements, the acquired height values will be different because of finite probe (tip) dimension. This deviation should depend on the particle and the tip shape (cone angle), on the particle coverage and their distribution within the layer

described by the pair correlation function. Although a precise calculation of this correction requires extensive Monte-Carlo type modeling, it significance can be estimated considering the two-particle geometry schematically shown in Figure S2. One can deduce that that a part of the surface becomes inaccessible to the tip if the average distance between the particle surfaces h_p becomes smaller than the critical distance h_p^* given by

$$h_p^* = d_p \tan\left(\frac{\alpha}{2}\right) + d_p\left[1 - \cos\left(\frac{\alpha}{2}\right)\right] \tag{S21}$$

where α is the cone angle of the tip (see Figure S2).

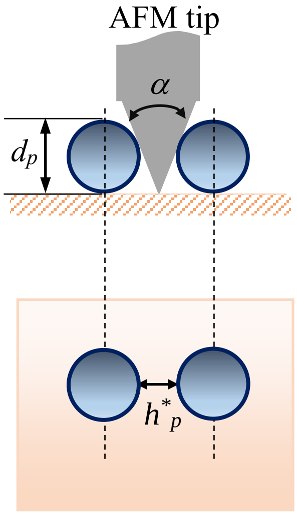

Figure S2. Schematic representation of the two spherical particle/AFM tip geometry pertinent to the large coverage limit for the critical surface to surface distance equal to h_p^*.

Considering that the average surface to surface distance between particles in the layer is given by the formula [8]

$$h_p = d_p\left[\left(\frac{\Theta_{mx}}{\Theta}\right)^{1/2} - 1\right] \tag{S22}$$

this postulate can be formulated as:

$$d_p\left[\left(\frac{\Theta_{mx}}{\Theta}\right)^{1/2}-1\right]<h_p^* \tag{S23}$$

where Θ_{mx} is the maximum particle monolayer coverage.

The inequality expressed by eq S23 can be converted to the explicit form:

$$\Theta<\frac{\Theta_{mx}}{(1+\overline{h_p})^2} \tag{S24}$$

where

$$\overline{h_p}=\frac{h_p^*}{d_p}=\tan\left(\frac{\alpha}{2}\right)+\left[1-\cos\left(\frac{\alpha}{2}\right)\right] \tag{S25}$$

As can be seen the critical distance between particles $\overline{h_p}$ (where the tip convolution effects star to appear) is independent of the particle size.

Considering that the typical cone angle of AFM tips is very small and varies between 15° and 30° eq S25 can be simplified to the following form

$$\overline{h_p}=\frac{h_p^*}{d_p}\cong\frac{\pi\alpha}{360} \tag{S26}$$

Therefore, for a sharp tip with the cone angle of 15° where $\overline{h_p}$ = 0.131, on can calculate from eq S24 that the convolution effects should appear if the particle coverage in the layer exceeds 0.43 (for the RSA process where Θ_{mx}= 0.547). For α = 30°, $\overline{h_p}$ = 0.262, which gives the critical particle coverage of the layer equal to 0.34.

If the normalized distance between particle surfaces becomes smaller than the above estimated values of $\overline{h_p}$, a part of the surface area becomes inaccessible to the tip, which instead moves around the particles. Considering that for two touching particles the maximum radius of the inaccessible surface area is equal to

$$r^*=\frac{1}{2}d_p\left[1-\cos\left(\frac{\alpha}{2}\right)\right] \tag{S27}$$

the normalized inaccessible surface area becomes:

$$S^* / \frac{1}{4}\pi d_p^2 \cong \frac{1}{\pi}\left[1-\cos\left(\frac{\alpha}{2}\right)\right]^2 \tag{S28}$$

On can calculated from eq S28 that even for the tip cone angle of 30° the normalized inaccessible surface area calculated from eq S28 is below 1%.

APPENDIX A (Supplement to Section 1)

In the section the following surface integrals are calculated for particles of various shape

$$\begin{aligned} h_1 &= \frac{1}{S_p}\int_s h(\mathbf{r}_s)\cdot d\mathbf{r}_s \\ h_2 &= \frac{1}{S_p}\int_s h^2(\mathbf{r}_s)\cdot d\mathbf{r}_s \\ h_3 &= \frac{1}{S_p}\int_s h^3(\mathbf{r}_s)\cdot d\mathbf{r}_s \\ h_4 &= \frac{1}{S_p}\int_s h^4(\mathbf{r}_s)\cdot d\mathbf{r}_s \end{aligned} \tag{A1}$$

Let us first consider the case of a particle in the form of a cap of an elliptic cross-section area (see Fig. A1).

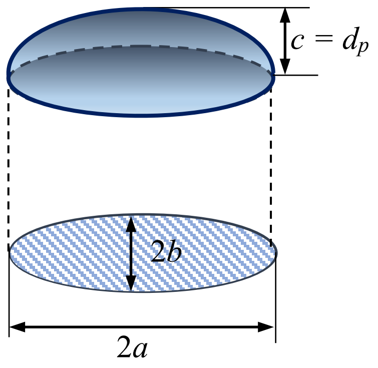

Figure A1. Schematic view of the ellipsoidal cap attached to a planar surface.

The cap shape is defined by the constitutive equation:

$$\frac{x^2}{a^2}+\frac{y^2}{b^2}+\frac{z^2}{c^2}=1 \tag{A2}$$

where (x,y,z) are local Cartesian coordinates with the origin at (0,0,0).

Analogously, the cap height expressed in this coordinate system is given by

$$h(x,y)=c\left[1-\frac{x^2}{a^2}-\frac{y^2}{b^2}\right]^{1/2} \tag{A3}$$

where $-a<x<a$, and $-b<y<b$. The cap cross-section area is $S_p=\pi ab$.

Considering this definition of the cap height and its symmetry, the double integrals defined by eq A1 can be expressed as follows

$$\begin{aligned}
h_1&=\frac{4c}{\pi ab}\int_0^a dx\int_0^{b\left(1-\frac{x^2}{a^2}\right)^{1/2}}\left(1-\frac{x^2}{a^2}-\frac{y^2}{b^2}\right)^{1/2}dy=\frac{4c}{\pi ab}I_1\\
h_2&=\frac{4c^2}{\pi ab}\int_0^a dx\int_0^{b\left(1-\frac{x^2}{a^2}\right)^{1/2}}\left(1-\frac{x^2}{a^2}-\frac{y^2}{b^2}\right)dy=\frac{4c^2}{\pi ab}I_2\\
h_3&=\frac{4c^3}{\pi ab}\int_0^a dx\int_0^{b\left(1-\frac{x^2}{a^2}\right)^{1/2}}\left(1-\frac{x^2}{a^2}-\frac{y^2}{b^2}\right)^{3/2}dy=\frac{4c^3}{\pi ab}I_3\\
h_4&=\frac{4c^4}{\pi ab}\int_0^a dx\int_0^{b\left(1-\frac{x^2}{a^2}\right)^{1/2}}\left(1-\frac{x^2}{a^2}-\frac{y^2}{b^2}\right)^{2}dy=\frac{4c^4}{\pi ab}I_4
\end{aligned} \tag{A4}$$

Substituting:

$$\begin{aligned}
Y&=\left(\xi^2-y^2\right)\\
\xi^2&=b^2\left(1-\frac{x^2}{a^2}\right)
\end{aligned} \tag{A5}$$

the integrals I_1-I_4 can be transformed to the following general form

$$I_q = \frac{1}{b^q}\int_0^a dx \int_0^{\xi} Y^{\frac{q}{2}}\, dy = \frac{1}{b^q}\int_0^a I_q^{'}(x)\, dx \tag{A6}$$

where q = 1, 2, 3, 4 and

$$I_q^{'} = \int_0^{\xi} Y^{\frac{q}{2}}\, dy \tag{A7}$$

Explicitly:

$$\begin{aligned}
I_1^{'} &= \int_0^{\xi} Y^{1/2}\, dy = \left[\frac{1}{2}y\left(\xi^2 - y^2\right)^{1/2} + \frac{1}{2}\xi^2 \arcsin\left(\frac{y}{\xi}\right)\right]\Bigg|_0^{\xi} = \frac{\pi}{4}b^2\left(1-\frac{x^2}{a^2}\right) \\
I_2^{'} &= \int_0^{\xi} Y\, dy = \left[\left(\xi^2 y - \frac{y^3}{3}\right)\right]\Bigg|_0^{\xi} = \frac{2}{3}b^3\left(1-\frac{x^2}{a^2}\right)^{3/2} = \frac{2}{3}b^3\left(1-\frac{x^2}{a^2}\right) \\
I_3^{'} &= \int_0^{\xi} Y^{3/2}\, dy = \left[\frac{1}{4}y\left(\xi^2 - y^2\right)^{3/2} + \frac{3}{8}\xi^2 y\left(\xi^2 - y^2\right)^{1/2} + \frac{3}{8}\xi^4 \arcsin\left(\frac{y}{\xi}\right)\right]\Bigg|_0^{\xi} = \frac{3\pi}{8}b^4\left(1-\frac{x^2}{a^2}\right)^2 \\
I_4^{'} &= \int_0^{\xi} Y^2\, dy = \int_0^{\xi}\left(\xi^4 - 2\xi^2 y^2 + y^4\right)dy = \frac{8}{15}b^5\left(1-\frac{x^2}{a^2}\right)^{5/2} \\
\xi &= b\left(1-\frac{x^2}{a^2}\right)^{1/2}
\end{aligned} \tag{A8}$$

Combining eq A8 with eq A6 one obtains the following integrals

$$\begin{aligned}
I_1 &= \frac{\pi}{4}b\int_0^a\left(1-\frac{x^2}{a^2}\right)dx = \frac{\pi}{6}ba \\
I_2 &= \frac{2}{3}b\int_0^a\left(1-\frac{x^2}{a^2}\right)^{3/2} dx \\
I_3 &= \frac{3}{8}\pi b\int_0^a\left(1-\frac{x^2}{a^2}\right)^2 dx = \frac{3\pi}{15}ba \\
I_4 &= \frac{8}{15}b\int_0^a\left(1-\frac{x^2}{a^2}\right)^{5/2} dx
\end{aligned} \tag{A9}$$

The I_2, I_4 integrals can be evaluated substituting

$$X^{1/2} = \left(a^2 - x^2\right)^{1/2} \tag{A10}$$

and considering that

$$\int X^{3/2} dx = \frac{1}{4} x\, X^{3/2} + \frac{3}{8} a^2 x X^{1/2} + \frac{3}{8} a^4 \arcsin\left(\frac{x}{a}\right) + const \tag{A11}$$
$$\int x^2 X^{3/2} dx = -\frac{1}{48}(-3a^4 + 14a^2x^2 - 8x^4)x\, X^{1/2} + \frac{1}{16} a^6 \arcsin\left(\frac{x}{a}\right) + const$$

In this way, combining eq A9 with eq A4 one obtains the final result for the cap

$$\begin{aligned} h_{1c} &= \frac{2}{3} c \\ h_{2c} &= \frac{1}{2} c^2 \\ h_{3c} &= \frac{2}{5} c^3 \\ h_{4c} &= \frac{1}{3} c^4 \end{aligned} \tag{A12}$$

These results are valid for arbitrary elliptic caps, obviously comprising the hemi-sphere.

Additionally, the results obtained for the caps can be generalized in a simple way to the case of a hybrid particle composed from on elliptic cylinder (disk) of the length *c* with an elliptic cap of the same height *c*. Thus, for such a particle $d_p = 2c$ and its height h_p can be expressed as

$$h_p = h_c\left(x, y\right) + c \tag{A13}$$

where $h_c\left(x, y\right)$ is the cap height.

Considering this, the h_1, h_2, h_3, h_4 topographical parameters for the hybrid particle can be expressed as

$$h_q = \frac{1}{S_p} \int_{S_p}\int \left(h_c + c\right)^q dx\, dy \tag{A14}$$

Explicitly,

$$h_1 = h_{1c} + c = \frac{4}{3}c = \frac{2}{3}d_p$$

$$h_2 = h_{2c} + 2h_{1c}c + c^2 = \frac{17}{24}d_p^2 \qquad \text{(A15)}$$

$$h_3 = h_{3c} + 3h_{2c}c + 3h_{1c}c^2 + c^3 = \frac{49}{80}d_p^3$$

$$h_4 = h_{4c} + 4h_{3c}c^3 + 6h_{2c}c^2 + +4h_{1c}c^3 + c^4 = \frac{43}{80}d_p^4$$

The topographical parameters were also derived for a hemi-cylinder of an elliptic cross-section area attached side-on to the surface (perpendicular to the surface) (see Figure A2).

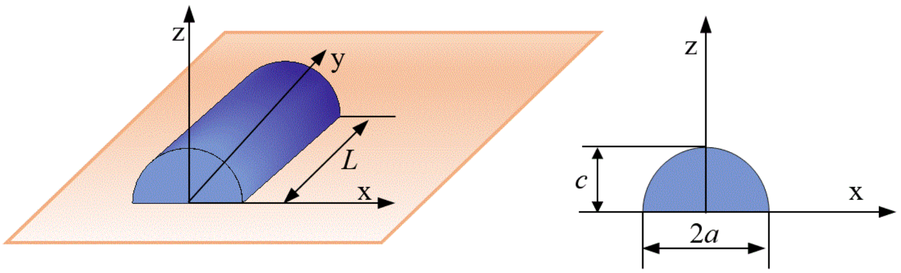

Figure A2. Schematic view of the hemi-cylinder attached to a planar surface.

In this case the h_q parameters are given by

$$h_q = \frac{1}{2aL} 2Lc \int_0^a \left(1 - \frac{x^2}{a^2}\right)^q dx \qquad \text{(A16)}$$

where L is the cylinder length.

Evaluating the integrals expressed by eq A16 one obtains

$$h_1 = \frac{c}{a^2}\int_0^a X^{1/2} dx$$

$$h_2 = \frac{c^2}{a^3}\int_0^a X\ dx \qquad \text{(A17)}$$

$$h_3 = \frac{c^3}{a^4}\int_0^a X^{3/2} dx$$

$$h_4 = \frac{c^4}{a^5}\int_0^a X^2 dx$$

where, a before: $X = a^2 - x^2$

Evaluating these integrals one obtains

$$h_1 = \frac{\pi}{4}c$$
$$h_2 = \frac{2}{3}c^2 \qquad \text{(A18)}$$
$$h_3 = \frac{3\pi}{16}c^3$$
$$h_4 = \frac{8}{15}c^4$$

Analogously as before, one can generalize these results to the hybrid particle composed of a parallelepiped of the rectangular cross-section 2*ac* and the height *L*, and the hemi-cylinder (cap) analyzed above.

Combining eq A18 with eq A15 the topographical parameters for such composite particle become

$$h_1 = \frac{1}{2} + \frac{\pi}{8}$$
$$h_2 = \frac{5}{12} + \frac{\pi}{8} \qquad \text{(A19)}$$
$$h_3 = \frac{3}{8} + \frac{15}{128}\pi$$
$$h_4 = \frac{83}{240} + \frac{7}{64}\pi$$

References

1. Hinrichsen, E.L.; Feder, J.; Jøssang, T. Geometry of random sequential adsorption. *J. Stat. Phys.* **1986**, *44*, 793–827.
2. Viot, P.; Tarjus, G.; Ricci, S.M.; Talbot, J. Random sequential adsorption of anisotropic particles. I. Jamming limit and asymptotic behavior. *J. Chem. Phys*. **1992**, *97*, 5212 –5218.
3. Evans, J.W. Random and cooperative sequential adsorption. *Rev. Mod. Phys.* **1993**, *65*, 1281–1329.
4. Adamczyk, Z.; Siwek, B.; Zembala, M.; Weroński, P. Influence of polydispersity on random sequential adsorption of spherical particles. *J. Colloid Interface Sci.* **1997**, *185*, 236–244.
5. Talbot, J.; Tarjus, G.; van Tassel, P.R.; Viot, P. From car parking to protein adsorption: an overview of sequential adsorption processes. *Colloids Surf. A*. **2000**, *165*, 287–324.
6. Sadowska, M.; Cieśla, M.; Adamczyk, Z. Nanoparticle deposition on heterogeneous surfaces: Random sequential adsorption modeling and experiments. *Colloids Surf. A*. **2021**, *617*, 126296-1 - 126296-9.
7. Adamczyk Z. Particles at interfaces: Interactions, deposition, structure. *Elsevier*. London, **2017**.
8. Adamczyk, Z.; Morga, M.; Nattich-Rak, M.; Sadowska, M. Nanoparticle and bioparticle deposition kinetics, *Adv. Colloid Interface Sci*. **2022**, *302*, 102630-1 – 102630-23.
